# Focus on Human Monoamine Transporter Selectivity. New Human DAT and NET Models, Experimental Validation, and SERT Affinity Exploration

**DOI:** 10.1021/acschemneuro.0c00304

**Published:** 2020-09-29

**Authors:** Gabriella Ortore, Elisabetta Orlandini, Laura Betti, Gino Giannaccini, Maria Rosa Mazzoni, Caterina Camodeca, Susanna Nencetti

**Affiliations:** † Department of Pharmacy, University of Pisa, Via Bonanno 6, 56126 Pisa, Italy; ‡ Research Center “E. Piaggio”, University of Pisa, Pisa 56122, Italy; § Department of Earth Sciences, University of Pisa, Via Santa Maria 53-55, 56100 Pisa, Italy

**Keywords:** SERT, DAT, NET, 3D-QSAR model, homology modeling, 4-phenylpiperidine

## Abstract

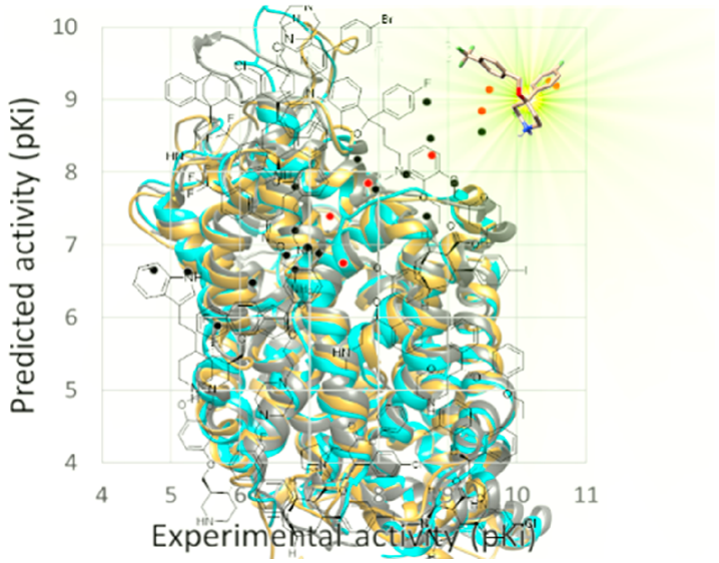

The most commonly used antidepressant drugs are the serotonin transporter inhibitors. Their effects depend strongly on the selectivity for a single monoamine transporter compared to other amine transporters or receptors, and the selectivity is roughly influenced by the spatial protein structure. Here, we provide a computational study on three human monoamine transporters, i.e., DAT, NET, and SERT. Starting from the construction of hDAT and hNET models, whose three-dimensional structure is unknown, and the prediction of the binding pose for 19 known inhibitors, 3D-QSAR models of three human transporters were built. The training set variability, which was high in structure and activity profile, was validated using a set of in-house compounds. Results concern more than one aspect. First of all, hDAT and hNET three-dimensional structures were built, validated, and compared to the hSERT one; second, the computational study highlighted the differences in binding site arrangement statistically correlated to inhibitor selectivity; third, the profiling of new inhibitors pointed out a conservation of the inhibitory activity trend between rabbit and human SERT with a difference of about 1 order of magnitude; fourth, binding and functional studies confirmed 4-(benzyloxy)-4-phenylpiperidine **20a–d** and **21a–d** as potent SERT inhibitors. In particular, one of the compounds (compound **20b)** revealed a higher affinity for SERT than paroxetine in human platelets.

## Introduction

The current generation of antidepressant drugs, both more effective and more tolerable than older antidepressants, acts predominantly by targeting the serotonin transporter (SERT).^1^ After a long period of selective serotonin reuptake inhibitors (SSRIs) predominance,^2^ with the introduction of drugs such as fluoxetine, citalopram, sertraline, and paroxetine, researchers focused on additional pharmacologic mechanisms.^3^ In the early 1990s, the serotonin-norepinephrine reuptake inhibitors were commercialized;^4^ in the first decade of the 21st century, there were incremental studies on serotonin-norepinephrine-dopamine reuptake inhibitors;^5^ and over the past decade, dual action inhibitors have emerged, which had very different sizes and scaffolds compared to pure reuptake inhibitors, while retaining good affinities for SERT.^6^ Among these are vilazodone, which combines 5-HT_1a_ partial agonism with SERT inhibition,^7^ and vortioxetine,^8^ which combines 5-HT_1a_ and 5-HT_1b_ partial agonism, 5-HT_7_ and 5-HT_3_ antagonism, and SERT inhibition. These various strategies provided the possibility of targeting residual symptoms, which were not well treated by SERT inhibition alone, and also reducing the side effects, such as sexual dysfunction, but at the same time, they introduced other side effects due to the action against multiple receptors.

In this context, the diversity of the structures able to inhibit SERT induced a deep curiosity for the transporter structure and the binding site location. On the other hand, the search for transporter-selective ligands requires the knowledge of the SERT structural requirements. Initially, due to the lack of a crystal structure, many attempts to construct a homology model based on the LeuT crystal structure were performed for rationalizing the affinity of so different drugs against SERT.^9−16^ The SERT models published before 2009 were usually constructed using LeuT as a template in its outward-occluded structure. After the crystallization of LeuT with a competitive inhibitor in its open-to-out conformation, the outward-open structures of the transporter were also studied.^13,14^ In these outward-open structures, the vestibular, usually denoted as S2 and recently as allosteric, and the substrate (S1) binding sites, which were separated in the occluded form through the Tyr176-Phe335 gate, were combined in one cavity. This was considered by several experts as the putative binding site of SSRIs, which are thought to stabilize the outward-facing conformations of SERT by preventing closure of the extracellular gate.^17,18^

**Chart 1:**
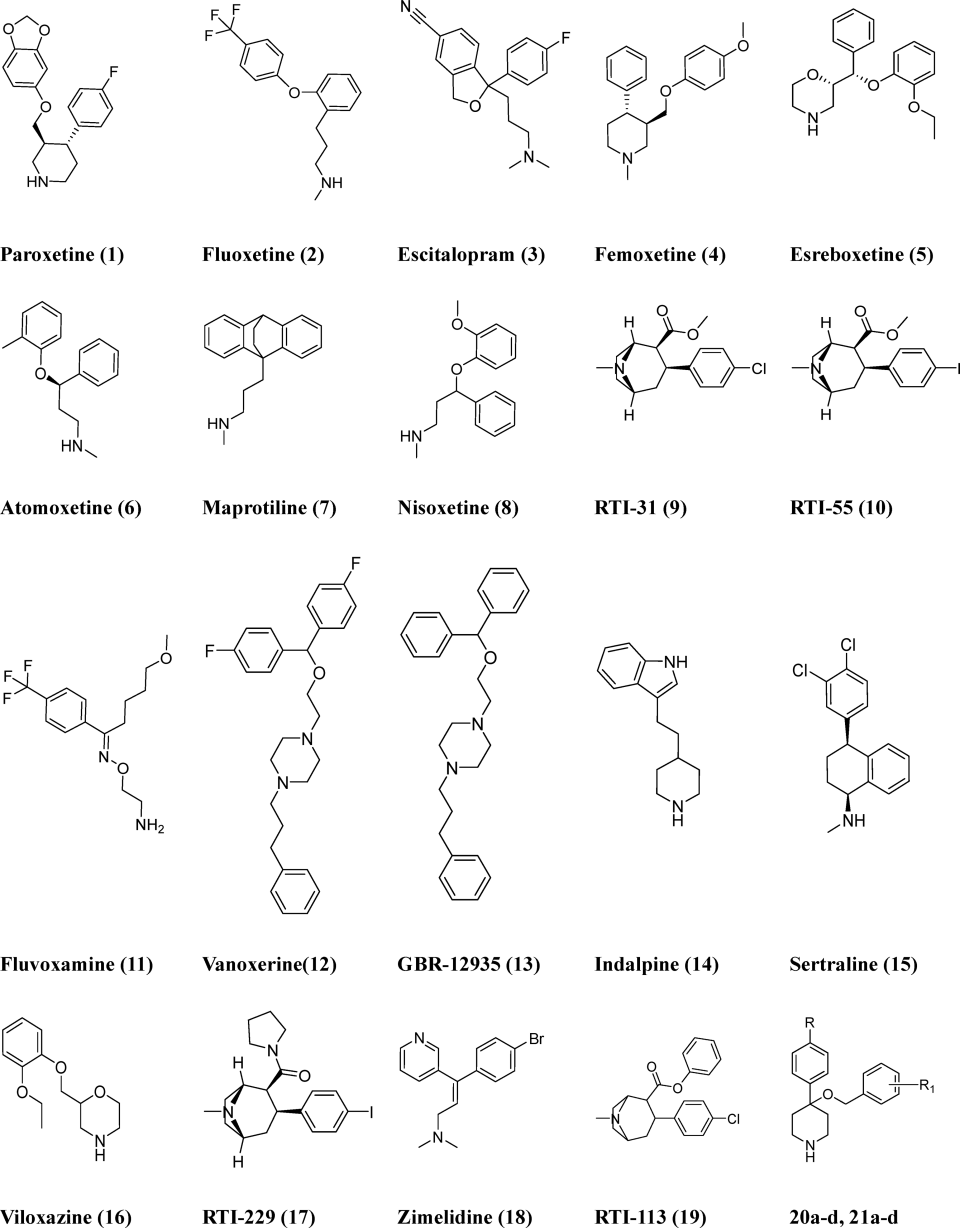
Chemical Structures of the Compounds Studied by Docking in hDAT, hNET, and hSERT Models and Used as a Training Set for 3D-QSAR Evaluations **(1–19)** and in-House Compounds Used as an External Test Set (**20a–d** and **21a–d**)

At the moment, 3D structures of dDAT and hSERT are available,^19^ and the structures have led to advancement in the study of the transporter interactions with their ligands and of the structural differences among SERT, DAT, and NET. Unfortunately, dDAT and hDAT share only 55% *homology* at the amino-acid level. This degree of structural similarity is not enough to directly extrapolate hDAT information from dDAT data. In fact, the homology between dDAT and hDAT is similar to that found for each human transporter related to the others (hDAT/hNET = 67%, hDAT/hSERT = 50%, hNET/hSERT = 53%). Another consideration relates to the transporter crystal structure resolution, which is ≥3 Å for all structures deposited in the Protein Data Bank (PDB) to date.^19^ This value could not guarantee correct folding and accurate side-chain rotamers. Furthermore, until 2019 hSERT was crystallized in an engineered form that contained a point mutation in a strategic position, namely Thr439Ser. This position represents an interaction point for several inhibitors, including escitalopram and paroxetine, and it is one of the main binding site differences between hSERT and hNET. As the 3D structure of NET is also lacking, it is difficult to elucidate the molecular basis of the transporter inhibition. Our aim is the construction and validation of human amine transporter models, for rationalizing the selectivity of known inhibitors and to highlight the structural differences in protein arrangement which are responsible for their different activities against hDAT, hNET, and hSERT. This goal is ambitious considering that a very small change in inhibitor substitutions can produce a weak difference in the activity against one transporter and a full order of magnitude variation in another one. Validated models could also be a good starting point for designing novel compounds and predicting new inhibitor potency. We therefore constructed the hDAT and hNET models and optimized the wild-type form of hSERT, using the successive cryo-EM structure of wild-type hSERT for comparison.^20^ In order to investigate the basis of selectivity for SERT and unveil a strategy for improving the potency of some interesting in-house piperidine derivatives, which show high affinity for SERT,^21,22^ we defined the biological profile of compounds **20a–d** and **21a–d** (Chart 1).

## Results and Discussion

### Construction of the Transporter Models

Our work aimed to elucidate the inhibitor selectivity for SERT and therefore was principally focused on the binding site structure. The investigation began with a BLAST^23^ search of hDAT and hNET sequences derived from the UNIPROT Web site,^24^ using the PDB database as a search set.^19^ The BLAST analysis of the sequence homology showed a similar identity and query coverage using the 3D structure of crystallized dDAT or hSERT as a template (see Table 1). The higher similarity between hNET and hDAT (67% of identity) was not preserved comparing hNET with dDAT (58% of identity), so there was no reason to choose just one preferred transporter as a template to construct hDAT and hNET models. We chose to perform a multitemplate modeling and use the structural information on both the crystallized transporters in the construction of our targets.

**Table 1:** Summary of BLAST Analysis Results

	resulting PDB accessions	
query	dDAT (11 structures)	hSERT (3 structures)
hNET	58–59% identity	53% identity
	87–89% query coverage	88% query coverage
	642–667 total score	603–606 total score
hDAT	55% identity	52% identity
	87–88% query coverage	87% query coverage
	619–636 total score	591–594 total score

The alignment of the human transporters on the dDAT and hSERT sequences (Figure S1) showed high consensus scores except for the EL3 region and unaligned N- and C-termini. Only the last helices, TM9 to TM12, showed some variability in the sequences with a consequent decrease of the consensus scores. However, such variability did not interfere with a good alignment of the transporters on the templates. The three-dimensional models of hNET and hDAT were generated using the MODELLER program,^25^ on the basis of the multialignment reported in Figure S1. MODELLER constructed the unaligned EL2 loop using the simulated annealing, preserving the strictly conserved disulfide linkage between two conserved cysteines of EL3: Cys180 and Cys189 of hDAT and Cys176 and Cys185 of hNET.^26^ The longest unaligned tract was the N-terminal chain, which is irrelevant in studying the binding of inhibitors to the transporter.

**Figure 1:**
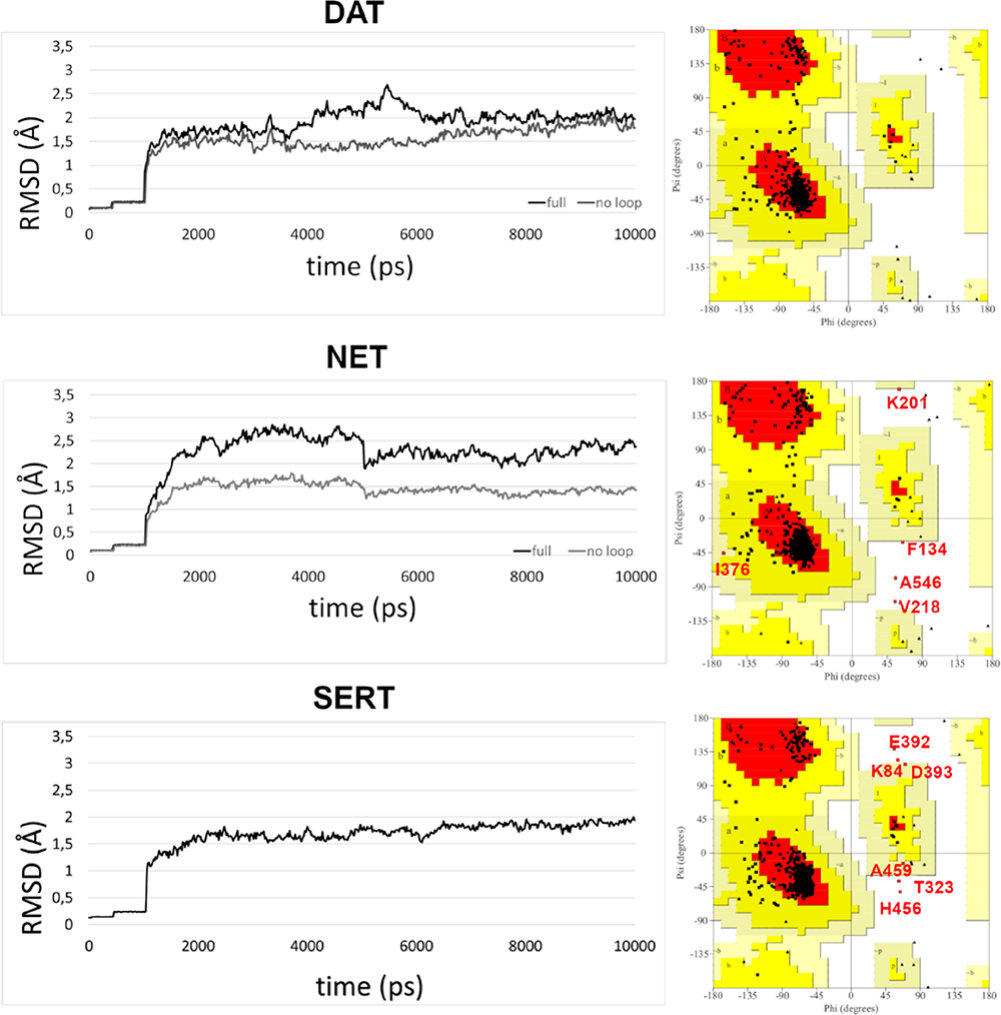
RMSD of alpha-carbons of the protein from the postheating coordinates. The dark colored line is the RMSD of the full sequence, while the gray colored line represents the RMSD of the structured region sequence.

The hSERT crystal structure (PDB code 5I6X) was just mutated in the four points engineered: Ala291Ile, Ser439Thr, Ala554Cys, and Ala580Cys. The models were refined by means of Molecular Mechanics (MM) and Dynamics (MD) calculations in a fully hydrated phospholipid bilayer environment and checked with PROCHECK^27^ (see the Methods section for details). The Ramachandran plots of hNET and hSERT models (Figure 1) showed four and six residues in disallowed regions, respectively. In hSERT, Lys84 was in the N-terminal region and Thr323 was at the end of EL3 loop, while Glu392 and Asp393 were localized in the EL4 loop, and His456 and Ala459 were in IL4. The four disallowed residues of hNET, Phe133, Lys201, Ile376, and Asp546, were situated in IL1, EL2, EL4, and EL6, respectively. They were all in the border regions, exposed to the solvent, far away from the binding sites.

The stability of the models was evaluated by calculating the root-mean-square deviation (RMSD) of the alpha carbons of the transporters along the trajectory (see in Figure 1 the postheating trajectory) from the starting model structures, hSERT, hDAT, and hNET. In all transporter plots, the effects of the first constraint’s relax after 400 ps and total relax of residues after 1000 ps were evident. Starting from a very refined structure, the higher stability of hSERT in comparison with the other transporters was also evident. In hDAT, the whole system showed only small fluctuations near 2 Å of RMSD during the simulation; in the range between 5 and 6 ns, the RMSD increment was due to the intracellular loop flexibility, as confirmed by the “no loop” (gray) plot. During the dynamic simulation, the hNET graph showed more fluctuations of about 2.5 Å of RMSD in the full sequence plot and of 1.5 Å in the plot restricted to the structured regions (gray plot). Between 2 and 5 ns of simulation, an instability concerning especially the folding of the intracellular tract of TM8 was registered. In general, all systems achieved an equilibrated structure.

The final model of hDAT showed an overall structure deviation of 2.8 Å (1.22 Å considering just the structured regions) with respect to the dDAT crystal structure (see Figure S2a).

In particular, TM5, TM12a, and IL10 presented a shift of the helices over the starting template which reached 2 Å of distance in some points. This is due to the degree of not conserved residues, which caused in the free dynamics simulation a rearrangement of some structured regions. As an example, in Figure S3 the IL5 loop is represented. The alignment target–template in this region was very poor, and the substitution Pro514 (dDAT) - Arg515 (hDAT) produced a different turn of the backbone. Moreover, the hydrogen bond between IL10 and IL6, involving residues Tyr337 and Asp509 in the crystal structure of dDAT, was disrupted in hDAT because of Tyr337 and Asp509 substitution with Phe338 and Gln510, respectively. The structured tract of IL10 shifted 2 Å.

The impact of the overall different packing in the binding site is reported in Figure S2b where the main unconserved residues are labeled. The binding site of hDAT resulted in being slightly larger than the dDAT one, in particular in the TM10 tract. The different conformation of the unwound region between TM6a and TM6b is most significant. In fact, the substitution of dDAT Pro323 with hDAT Val324 turns the entire tract of the backbone in such a way to direct hDAT Phe326 toward Ile484. This arrangement of the backbone and side chains probably concurs to the shift of TM10 and the enlargement of the binding site.

Predictably, the situation of hSERT, which started from a 3D structure analogue to the targeted one, was very different. The overall deviation between the initial and refined model (see Figure S2c) was 1.8 Å (1.06 Å considering just the structured regions). In the binding site (Figure S2d), there was a diffuse but slight change of the backbone and side chain arrangement partially due to the substitutions generated for the construction of the transporter in the wild-type form (e.g., the Ser439Thr mutation). An example is the ribbon shift near the piperonylic moiety of paroxetine.

**Figure 2:**
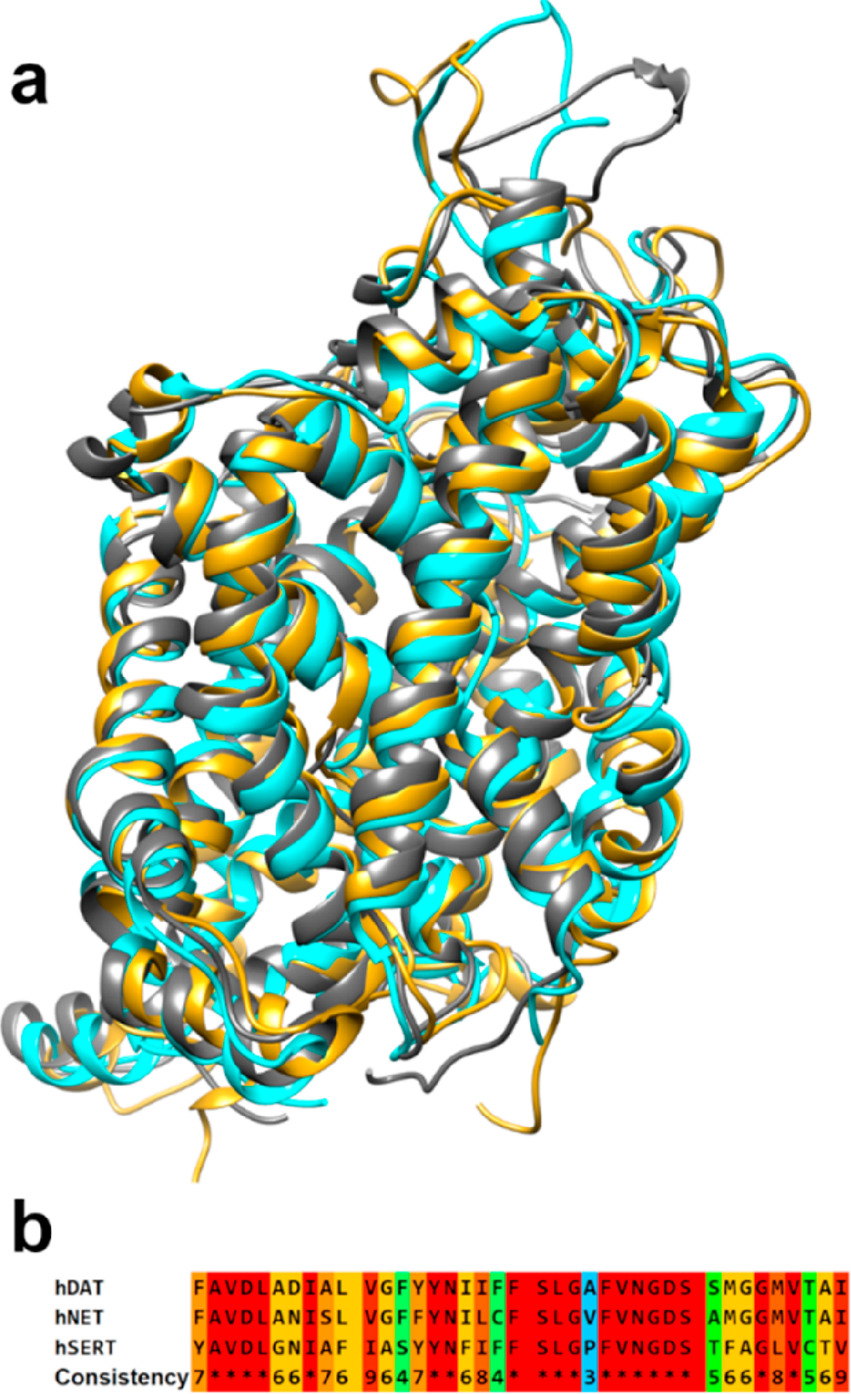
a) Superimposition of hDAT (cyan), hNET (gold), and hSERT (gray) models and b) sequence alignment of the binding site residues: in red are highlighted the identical regions, while conservative substitutions are highlighted in scale from cyan to orange.

The three final models are reported in Figure 2a, which shows the structured regions are quite superposed with an analogue RMSD of the C alpha of all transporters of 1.2 Å. Obviously, the largest variability is due to loop and terminal regions.

Small differences in helix windings are detectable in many regions. In particular, there are many fluctuations in TM10 and TM12 because of the significant variability of EL6 conformation, which strongly interacts with TM10, and the presence of a helix turn in TM10. Anyway, the effects of these fluctuations are not appreciable in the binding site.

**Figure 3:**
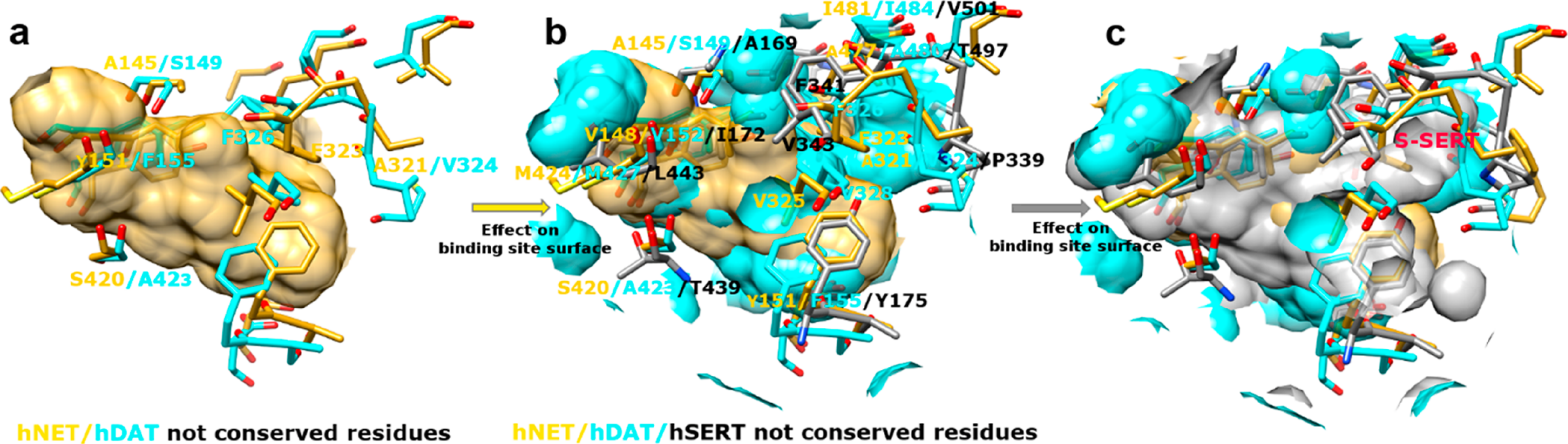
Effect of the substitutions on the size and shape of the three transporter binding sites: a) binding site surface of hNET; b) hDAT (cyan colored) superposed on hNET; and c) hSERT superposed on both the other transporters. Nonconserved residues are expressed, together with some critical conserved residues.

The binding site amino acid composition shows about 50% conservation and main semiconservative mutations across the transporters with only three nonconservative mutation points (see Figure 2b). This degree of modifications inside the binding site region produces a different shape and volume distribution of the cavity. In particular, some mutation points strongly influence the accessibility of specific microdomains. As reported in Figure 3, substitutions such as Ala145/Ser149, Tyr151/Phe155, and Ala321/Val324 between hNET and hDAT or the different conformations of hNET Phe323 and hDAT Phe326 create some supplementary cavities (cyan zones in Figure 3b) in the hDAT binding site. Further modifications in hNET/hDAT/hSERT, such as Ala145/Ser149/Ala169, V148/V152/I172, M424/M427/L443, S420/A423/T439, and Y151/F155/Y175, produce a diffuse but modest enlargement of the principal cavity. The different conformations of the unwound region between TM6a and TM6b were already discussed concerning dDAT and hDAT. In this region, there is high homology between hSERT and dDAT and high conservation between hNET and hDAT, which cause a pocket widening in the zone labeled as S-SERT in Figure 3c. The principal reason for the different conformation of this turn in hSERT containing the main chain of Phe341 is the presence of Pro339 substituted by Ala and Val in hNET and hDAT, respectively. Ile481/Ile484/Val501 and Ala477/Ala480/Thr497 mutations further contribute to the creation of the S-SERT cavity, and the last one is also responsible for a different polarity of the microdomain.

### Docking of Known Ligands

In order to test the ability of hSERT, hDAT, and hNET models to predict both potency and selectivity of monoamine transporter inhibitors, such models were used for the docking of well-known ligands. The attempt to find in the literature homogeneous competitive displacement data for these inhibitors was not easy at all. For many inhibitors, the results of reuptake experiments were available instead of displacement data. Moreover, the *K*_i_ or IC_50_ values were often determined on tissues/cells from different species, affecting the collection of homogeneous data. However, as reported from Han and co-workers,^28^ it is possible to consider the test results performed in rodent and human tissues as comparable since human and mouse transporters show a similar sensitivity to tested drugs.

From the literature we collected the *K*_i_ values of 19 compounds, which were chosen in order to explore different chemical structures and selectivity profiles (compounds **1**–**19**, Chart 1) among TCAs (tricyclic antidepressants), SSRI, SNRI (selective norepinephrine reuptake inhibitors), and SDRI (selective dopamine reuptake inhibitors).

As in our previous study,^29^ docking of compounds **1**–**19** was performed by using the GOLD program.^30^ In addition, a double check of docking poses was realized through Flapdock.^31^ These two programs use very different methodologies for the pose prediction: the first one calculates the solvent accessible surface of each atom in the defined binding site, assigning potential donor and acceptor fitting points. Each trial ligand docking is generated through a genetic algorithm by a least-squares fit of mapping points, and one or more protein side chains can be treated as flexible. The FLAP program uses fingerprints, derived from the GRID Molecular Interaction Fields (MIFs).^32^ Ligand conformers are generated and then scored inside the pocket using the GRID MIF similarities (describing hydrogen-bonding interactions, hydrophobic interactions, and shape) and additional energy terms. In this work, the same main parameters were selected for calculations with both programs (see the Methods section for details). Exceptions are in regards to peculiar features of each program, as the flexibility of some selected side chains during the GOLD docking, or the accuracy of the pocket surface mapping due to the variety of available GRID probes in Flapdock.

**Figure 4:**
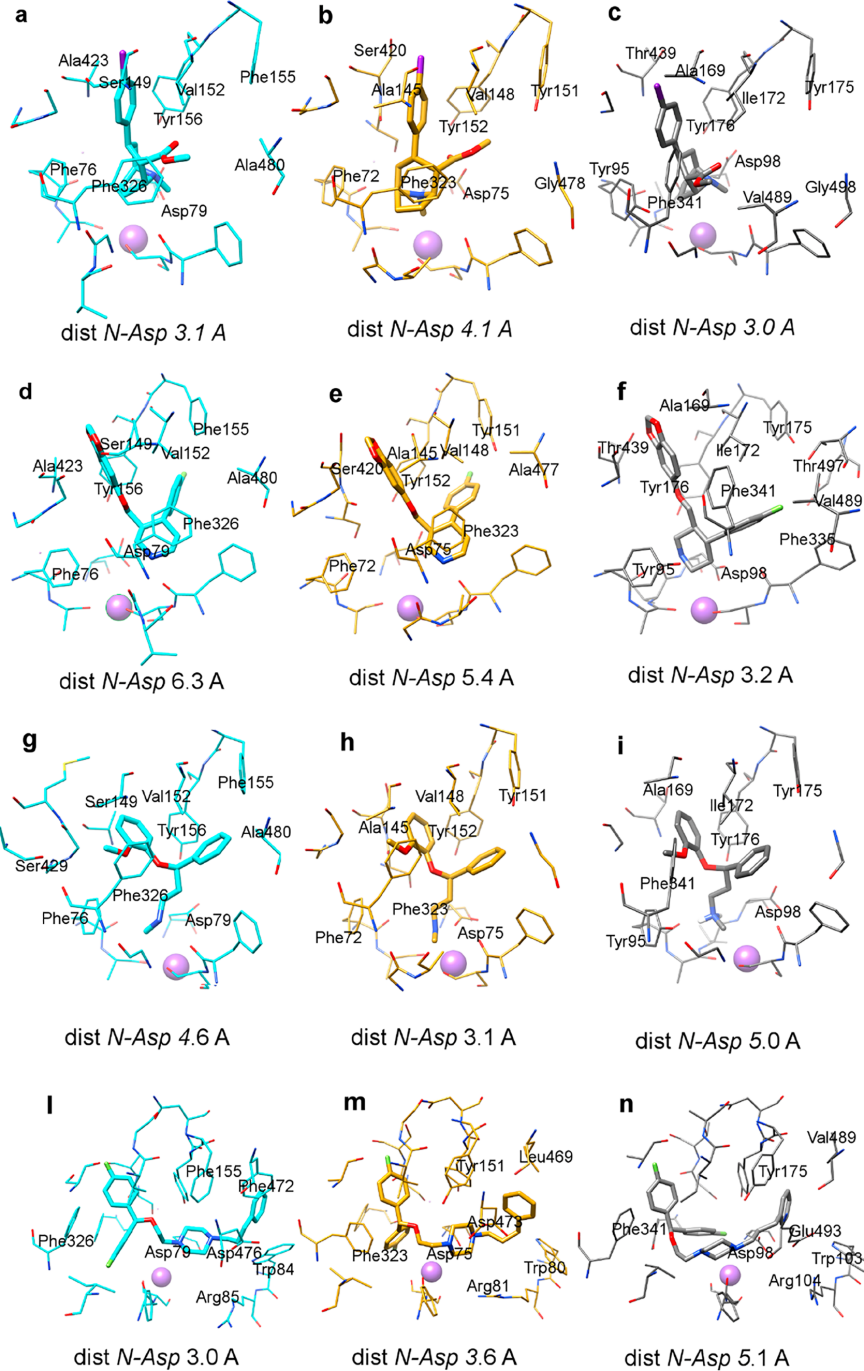
Docking of some classical transporters inhibitors: RTI-55 (a,b,c), nisoxetine (d,e,f), paroxetine (g,h,i), vanoxerine (l,m,n) in hDAT (cyan colored), hNET (gold colored), and hSERT (gray colored) models.

Figure 4 reports the docking predicted through the GOLD program^30^ of four crucial compounds, the not selective DRI RTI-55 (**10**), the SNRI nisoxetine (**8**), the SSRI paroxetine (**1**), and the SDRI vanoxerine (**12**), in hNET, hDAT, and hSERT.

The region occupied by RTI-55 (**10**), nisoxetine (**8**), and paroxetine (**1**) is the same; only vanoxerine (**12**), due to the larger size of the molecule, explores the adjacent space engaging interactions with further residues. All compounds place the protonated amine near the central TM1 flexible hNET Asp75, hDAT Asp79, and hSERT Asp98 although with a variable distance between 3 and 6 Å. The strength of this interaction seems to be correlated to the range of inhibition, although it is not the only requirement.

#### GOLD Docking of RTI-55

The RTI-55 pose in hNET and hDAT is very similar. A small difference in inclination of the phenyl group is due to the interaction of iodine, which prefers the not conserved hDAT Ser149 (hNET Ala145) and hNET Ser420 (hDAT Ala423) for halogen bonding. Furthermore, a rotation of about 60° of the tropane moiety is detected, due to the hindrance of hNET Tyr151(hDAT Phe155), whose hydroxyl group causes a shift of the ester group toward Tyr152. Both these differences produce a distancing of Asp79 in hNET and a weakening of lipophilic stabilization of the methoxy group due to Val152, Phe326, and Ala480 in hDAT. In hSERT, the ligand assumes a different orientation, due especially to the presence of Ile172 (Val in the other transporters), which precludes the insertion of the iodophenyl ring in the same cavity of hDAT and hNET. Moreover, the presence of Tyr95 instead of the hDAT and hNET Phe shifts the tropane moiety toward Asp98. A rotation of about 30 degrees avoids the clash with Ile172 and directs the methoxyl group in a region accessible just in hSERT (S-Sert in Figure 3c), due to the “downward” conformation of Phe341, which is not equivalent to the ones of hDAT Phe326 and hNET Phe323. The swinging conformation of this residue was highlighted in the crystallographic structures of dDAT and hSERT and was stable during the molecular dynamics simulation of the three transporters. The final structure showed a similar conformation for this amino acid in hDAT and hNET, analogous to the dDAT one; the original “downward” conformation of Phe341 in the hSERT crystal structure was also retained during the simulation. Some plasticity of this residue was already discussed for dDAT crystallization,^33^ thus emerging that Phe325 rotates inward to maintain edge-to-face aromatic interactions with different scaffolded cocrystallized ligands (tropane-based RTI-55 and cocaine, nortriptyline and nisoxetine). In the hSERT crystallized complexes, Phe341 did not show the same plasticity. Just in the last structures cocrystallized with sertraline and fluvoxamine this amino acid assumed an alternative conformation to the “downward” one of paroxetine and escitalopram complexes, which is different anyway from the many conformations detected for Phe325 in dDAT.

No information about the pose of tropane-derivatives in hSERT was known since it was only cocrystallized with citalopram, paroxetine, sertraline, and fluvoxamine. To test the possible correlation between Phe341 conformation and the nature of the cocrystallized ligand, we performed a molecular dynamics simulation on the theoretical hSERT-cocaine complex using the same procedure and the same cocaine pose described in the Methods section for the construction of the other transporter models. Starting from some steric engagement between Ile172 and the ligand, the complex evolved toward a stable structure, which showed a value of RMSD between the starting and ending conformation of Phe341 and Ile172 and cocaine of 0.4, 0.1, and 0.7 Å, respectively. The analysis of the molecular dynamic simulation suggests that the “downward” conformation of Phe341 in the hSERT-cocaine complex was dependent on the proximity of the unconserved Ile172, rather than on the nature of the complexed ligand. The slightly different pose of RTI-55 in hSERT with respect to the other transporters allows anyway a good interaction with Asp98 and a disposition of the iodophenyl moiety in the cavity delimited by Tyr95, Ala169, Ile172, and Phe341. These last two residues are also responsible, together with Val489, for the lipophilic stabilization of the methoxy group. The role of the carbonyl is not clear, which is not involved in particular interactions with the binding site.

A similar trend was predicted for RTI-31, while for RTI-229 and RTI-113 (see Figure S4) the bulkier substituents provoke a reversed docking in hNET, pointing the halogen toward Ala477 and disclosing the amine to Asp75. This result is not the only one suggested by the GOLD program, but it is the best scored. A similar pose to the ones assumed in hDAT and hSERT was generated with a lower score, showing a minor stability of the complex, probably due to the smaller cavity of hNET. In hDAT, the halogenated ring of RTI-113 and RTI-229 is superposed on the RTI-55 one, the phenoxy and pyrrolidine rings find a good location between unconserved Phe155 and Phe320, and the protonated amine lies at 3.4 Å from Asp79. In hSERT, a small deviation of the ligands due to the hindrance of unconserved Ile172 and Tyr175, in place of hDAT Phe155, shifts the ligand about 2 Å increasing the distance between Asp98 and the amine to 4.2 Å and reducing the stabilization of phenoxy and pyrrolidine rings.

#### GOLD Docking of Paroxetine

Paroxetine (**1**) occupies the same region in hDAT and hNET and is rotated in hSERT. However, the piperonylic moiety shows the same pose in all transporters. On the contrary, as for the RTI-55 docking, the fluorophenyl ring occupies two different cavities. In hDAT and hNET, the ring fills the same cavity, which is instead modified by Ile172 presence in hSERT. In hDAT, the piperonylic moiety interaction with unconserved Ser149 (hNET Ala145) and the Phe155 presence on the other side of paroxetine instead of a Tyr produce a shift of about 1 Å with respect to the pose of the same compound in hNET leading to an increment of the distance from Asp79. In hSERT, as in the 5I6X complex of PDB, this distance is about 3 Å. The insertion of the fluorophenyl ring in the cavity delimited by Ile172, Phe335, Phe341, and Val489 and the unconserved Thr497 guarantees a high lipophilic stabilization and a small quite polar surface able to receive, as in the crystallographic structures, the fluorine of paroxetine and also the cyano group of escitalopram. The docking of paroxetine in the *wt*-hSERT model is superposed to the crystallographic one, in spite of the small deviation in binding site arrangement due to Thr439Ser mutation.

#### GOLD Docking of Nisoxetine

The docking pose of nisoxetine (**8**) is also very similar to the one reported for the same compound in dDAT even though alternative dispositions characterized by similar scores were calculated by GOLD in all transporters. In particular, the methoxyphenyl ring could interchange the position with the unsubstituted one or rotate in such a way to put the methoxy chain toward the conserved Tyr (hDAT Tyr156, hNET Tyr152, and hSERT Tyr176). In hDAT and hNET, the nisoxetine disposition is analogous, but the presence of Ser149 and Phe155 in hDAT instead of Ala145 and Tyr151 of hNET produces a shift of the ligand, which weakens the interaction with hDAT Asp79. As already discussed for the 4XNU crystal structure (nisoxetine-dDAT complex),^34^ the pocket surrounding the methoxy chain in hDAT is quite polar with Ser149 and Ser429 in place of Ala145 and Ala426 of hNET. These serines are expected to have less favorable interactions with nisoxetine. In hSERT, the bulky Ile172 in the middle of the cavity partially clashes with the biaromatic system of nisoxetine, moving the protonated amine at a distance of 5 Å from Asp98. Interestingly, in all transporters, the flexible Phe (hDAT Phe326, hNET Phe323, and hSERT Phe341) almost assumes the same conformation, which is different from the starting position in hDAT and hNET and is able to stabilize the methoxy group. The congeners atomoxetine and esreboxetine have similar poses in hDAT and hNET with respect to nisoxetine (data not shown). In hDAT, the longer ethoxy chain of esreboxetine protrudes toward the unconserved Ser149 and Ser429 without appreciable effects on the protonated amine position. The polar serine environment is less favorable for the stabilization of the ethoxy moiety. In hNET, this portion of esreboxetine occupies the same lipophilic region as nisoxetine, but the rigid morpholine shifts in such a way to decrement the interaction with Asp75. In hSERT, the docking calculations predicted a different pose for esreboxetine with respect to atomoxetine and nisoxetine, giving the reversed pose as a lower scored pose, which puts the ethoxyphenyl toward Thr497. Actually, this kind of disposition is frequently predicted in all transporters with slightly lesser scores.

#### GOLD Docking of Vanoxerine

Different from compounds described above, there is no clear information about the possible pose of vanoxerine (**12**) in the transporters. It is one of the “atypical” DAT inhibitors possessing *in vivo* and *in vitro* effects, which are distinct from those of standard DAT inhibitors, such as cocaine. Atypical DAT inhibitors promote a longer-lasting increase in extracellular dopamine without any abuse potential.^35^

Both size and shape of vanoxerine are very different from those of the other inhibitors. Some mutational studies on hDAT revealed that the substitution of Trp84 with a Leu was related to an affinity decrease for diphenylmethoxy compounds such as vanoxerine, in contrast to the increment of *K*_app_ for tropane inhibitors.^36^

This evidence reinforced the previous hypothesis that “atypical” DAT inhibitors, like benztropine (BZT) and its diphenyl ether analogs (similar to vanoxerine), could stabilize the inward-facing conformation.^37^ In contrast, Cys accessibility results and molecular dynamics simulations suggested that aryltropane analogs can bind DAT and stabilize its outward-facing conformations like cocaine, yet producing effects that differ from those of cocaine.^38^ In addition, some diphenylmethoxy derivatives also seem to prefer an outward-open DAT conformation.^39^ In this context, the only experimental evidence is the probable involvement of Trp84 in vanoxerine binding stabilization. Our docking results show a binding pose filling the whole cavity and the propylphenyl chain of vanoxerine which protrudes toward the extracellular side. In hDAT, the monophenyl cap strongly interacts with Trp84 through an aromatic stacking strengthened by the presence of unconserved Phe472 and Phe155. This aromatic environment is unique for hDAT due to Phe472 substitution with the aliphatic residues Leu469 and Val489 in hNET and hSERT, respectively. Furthermore, in hSERT, the Trp103 region is less accessible for the presence of Glu493, which interacts with Arg104 occluding the cavity. In hDAT and hNET, the substitution of Glu493 with the shorter Asp476 and Asp473 opens the binding site toward the extracellular side making too long the distance toward the Arg residue. In hDAT, this assessment also produces a very good π–π interaction of the diphenoxy tail with Phe326 and a distance of 3 Å between the protonated amine and Asp79.

#### GOLD Docking of Other Known Inhibitors

The binding cavity is also preserved for other inhibitors, and the recent crystallization of fluvoxamine and sertraline in hSERT (pdb codes 6AWP and 6AWO) confirmed the pose predicted by our docking in the wild-type model. For fluoxetine and sertraline which have been already crystallized in LeuBAT, the pose predicted in hSERT is similar to the one in the LeuBAT complex crystal structure. Generally, all these hSERT selective inhibitors, such as escitalopram, show small deviations in the position assumed in the three transporters. Anyway, in hNET, they are affected by the presence of the shorter Asp473 instead of hSERT Glu493, which changes the shape of the region near Tyr151. For fluoxetine, fluvoxamine, and escitalopram, a reversed pose, which directs the fluorine or bulkier trifluoromethyl group between Tyr151 and Ala477, was also generated obtaining a similar score in hNET.

All small mutations disseminated along the binding site, which have been until now described, affected the binding of maprotiline (see Figure S5a). Since this drug is unable to plastically adapt to the binding site for its high rigidity, it yields a different orientation of the aminic chain. In hSERT, the presence of the longer Ile172 side chain prevents the insertion of the ligand in the usual orientation of the aminic chain toward Asp98.

With regards to indalpine (see Figure S5b), it occupies a half site sharing the position of the piperonylic group of paroxetine. Only in hSERT the cavity is large enough to direct the indole nitrogen toward Thr439. In this conformation, the piperidine well exposes the protonated amine to Asp79. In hDAT, in spite of the good stabilization of the indole through the unconserved Ser149, the ligand conformation cannot allow the interaction with Asp79. A similar orientation is calculated for indalpine in hNET. Zimelidine (Figure S5c), another hSERT selective inhibitor, occupies almost the same region of RTI-55 (see Figure 4a–c for comparison) but shows a different rigidity degree of the aromatic caps due to the geminal substitution on a double bond. Both in hDAT and hSERT, the propenyl linker directs the aminic cap 1.5 Å further away from Asp in comparison with RTI-55. In hNET, analogous to the bulkier RTI-229 and RTI-113, the halogenated aromatic cap prefers the insertion toward Ala477 showing a reversed docking mode, which prevents any interaction with Asp75.

#### FLAP Docking

**Figure 5:**
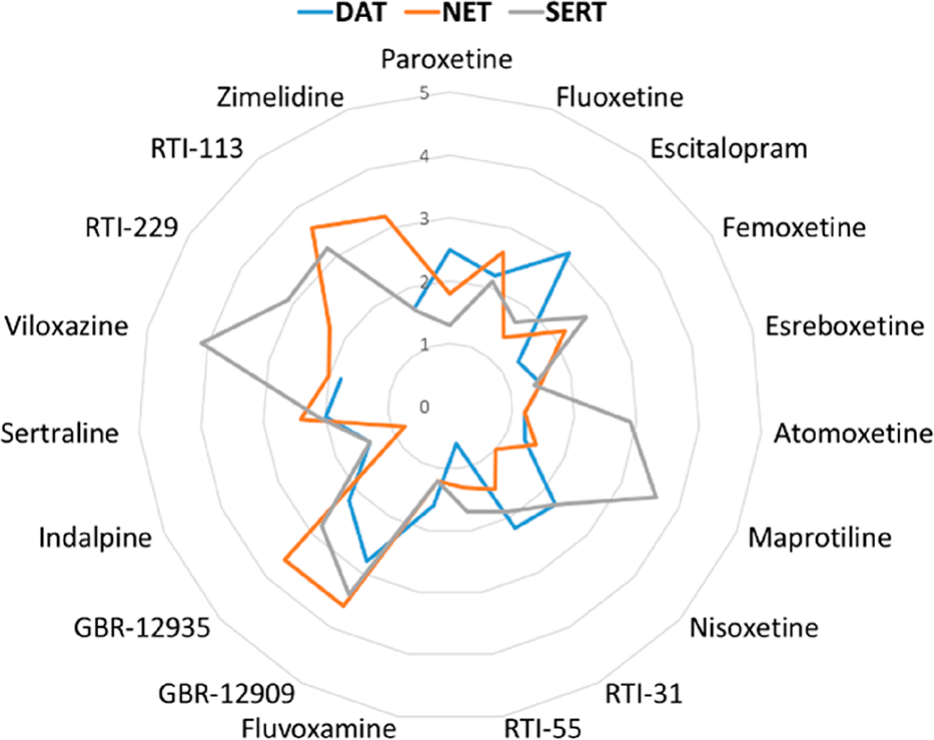
Comparison between the results of docking performed using GOLD and Flapdock programs. The RMSDs between the poses predicted for compounds **1**–**19** in the three transporters are reported.

Flapdock results have a trend very similar to the GOLD ones. Generally, the interaction with the central Asp (hNET Asp75, hDAT Asp79, and hSERT Asp98) is retained, and for many ligands reversed orientations are predicted in the same calculation with very similar scores. In Figure 5, a summary of the deviation between GOLD and Flapdock results, expressed as RMSD, is reported.

Most of the poses predicted through Flapdock show an RMSD less than 2.5 with respect to the GOLD predictions. Higher values are observed for GBR-12909 and GBR-12935, due to their high degree of freedom, and for viloxazine and maprotiline in hSERT. For these less bulky ligands, the docking is probably more conditioned by the Phe335 conformation, which is fixed in the Flapdock calculation. The only aromatic portion of these ligands prefers to align with the piperonylic moiety of paroxetine leaving the second cavity (also shaped by Phe335) empty. The bulkiest ligands have no alternative possibilities and fill both cavities. In the GOLD calculation, the flexibility of Phe335 allows a crude remodeling on-the-fly of the pocket. Similar to the GOLD results for RTI-113, RTI-229, and zimelidine, Flapdock emphasizes the prediction in hNET of two reversed poses with very similar scores retaining the ionic interaction with Asp79.

### Docking and Activities of Compounds **20a–d** and **21a–d**

**Chart 2:**
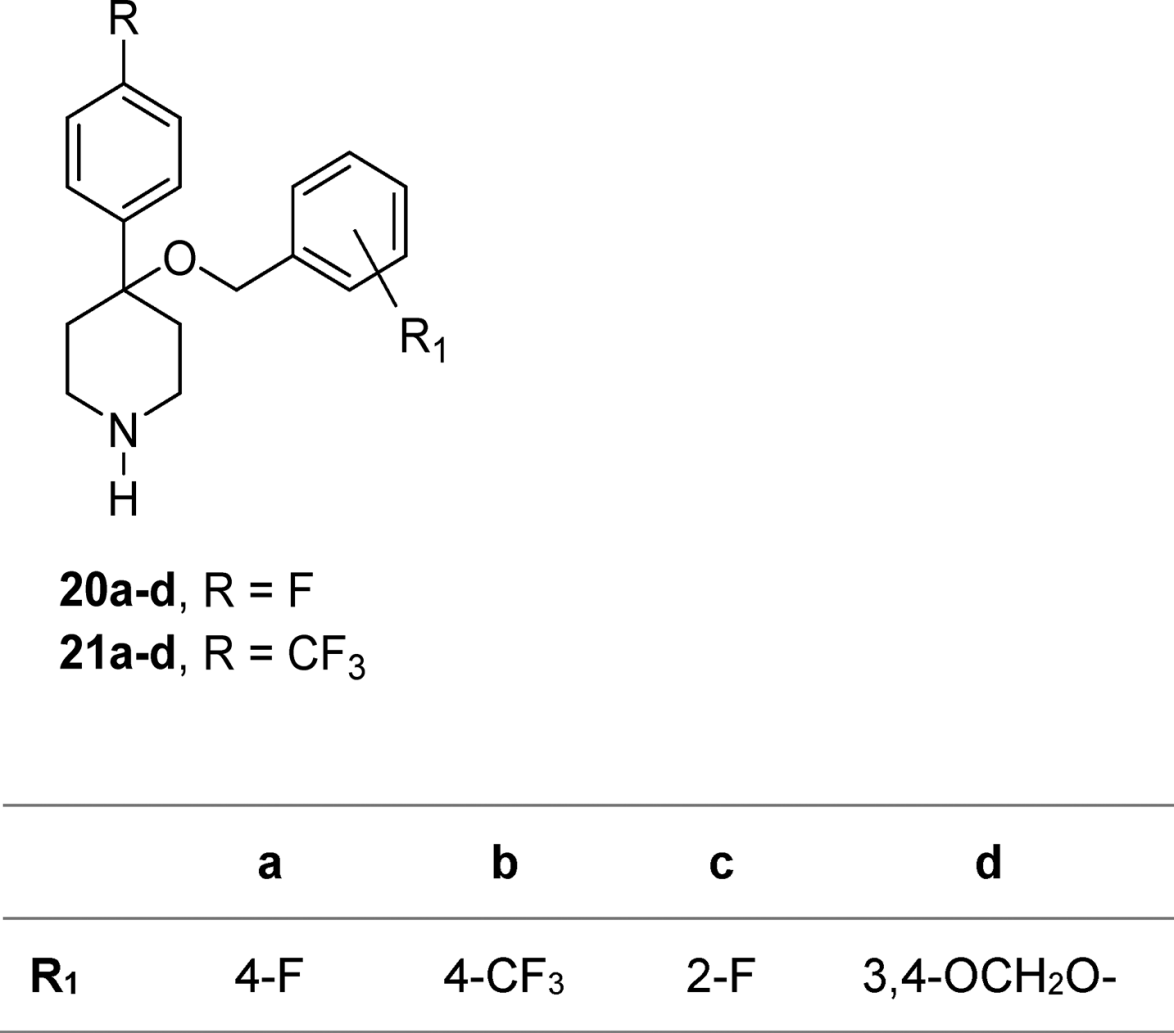
In-House Piperidine Derivatives **20a**–**d** and **21a**–**d** Used as an External Test Set

Piperidine derivatives **20a–d** and **21a–d**, which were designed in our laboratory and partially characterized as SERT inhibitors,^21,22^ are reported in this work to complete their profiling and are also used as the test set for our 3D-QSAR model (see Chart 2). They share principal features with paroxetine, such as the protonated piperidine, the methoxy spacer, and two aromatic rings at the ends of the molecule. Compounds **20d** and **21d** also share the presence of the piperonylic moiety with paroxetine.

However, the position of the substituents is different, and, in particular, the geminal substitution on piperidine represents an interesting feature leading to a slightly constrained conformation. Affinities of compounds **20b**, **20d**, **21b**, and **21d** toward the serotonin transporter have been already reported to be in the picomolar range^21,22^ overlapping that of paroxetine. Moreover, they revealed significantly lower affinity toward the dopamine transporter, thus indicating a higher selectivity of such compounds. In this paper, we tested the effect of 4-F and 2-F substitutions on their scaffold enlarging the set with analogues **20a**, **20c**, **21a**, and **21c**. Furthermore, we completed the affinity profile of these compounds adding the results of NET binding displacement assays.

**Figure 6:**
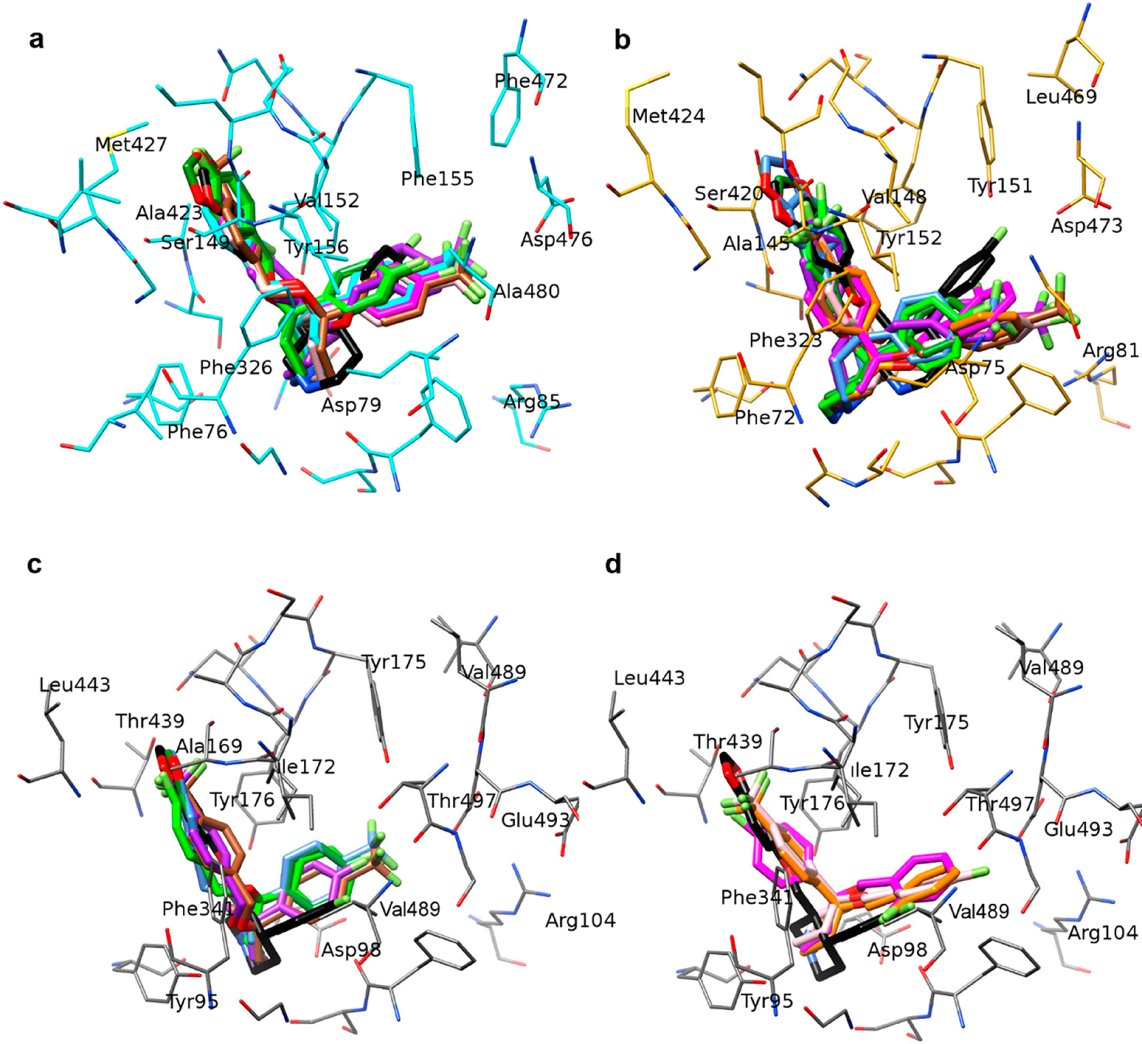
Docking of compounds **20a**–**d** and **21a**–**d** and paroxetine (black colored) in hDAT (a) and hNET (b); c) docking of compounds **20a** (light green), **20b** (purple), **20d** (forest green), **21b** (brown), and **21d** (light blue) and paroxetine (black) in hSERT; and d) docking of compounds **20c** (magenta), **21a** (pink), and **21c** (orange) and paroxetine (black) in hSERT.

In Figure 6, the poses calculated for compounds **20a–d** and **21a–d** are reported in comparison with the one of paroxetine since it is the most similar inhibitor. In all transporters, they occupy the same region. The piperonylic moiety of compounds **20d** and **21d** is superposed on the same moiety of paroxetine in all complexes, but the piperidine ring and especially the second aromatic moiety are disclosed to the paroxetine in hDAT and hNET. Instead of the interaction with Phe155 and Tyr156 of hDAT, or Tyr151 and Tyr152 of hNET, the second aromatic ring (in particular for the bulky trifluoromethyl derivatives **20b**, **21a**, and **21b)** occupies the region near Asp476 and Asp473 with the worst stabilization due to the high polarity of the cavity included between the Asp residue and an Arg residue (Arg85 and Ar81). This arrangement is not consistent with the hSERT binding cavity where the presence of Tyr95 instead of the Phe residue of hDAT and hNET shifts the piperidine ring toward Asp98 and Tyr176. In this pose, the R substituent is not inserted in the polar region between charged amino acids, also because the longer side chain of Glu382 makes a bridge with Arg104 closing the cavity. It occupies the same area of the paroxetine fluorine, between Thr497 and Val489, in the region already labeled as S-SERT in Figure 3c. As if there was a general rule, all compounds direct the bulkiest end toward Thr439 in hSERT; just the ortho-substituted phenyl moiety of compounds **20c** and **21c** prefers the opposite cavity toward Thr497. Therefore, in hSERT, all compounds arrange the methoxyaromatic chain toward Thr439 (Figure 6c), like paroxetine, except for compounds **20c, 21a**, and **21c**, which direct it toward Thr497. The interaction of compounds **20a–d** and **21a–d** with the central Asp residue is of the same extent of paroxetine, but the higher rigidity of their scaffold, in particular in compounds with bulkier substituents, leads to the worst stabilization of one aromatic end in hDAT and hNET. In hSERT, the shape and size of the S-SERT region allow a better stabilization of these compounds, especially if the ligand substituent is sterically able to fit the cavity near Thr439. The docking poses are in good correlation with the high affinity of compounds **20b**, **20d**, **21b**, and **21d** for hSERT and with a 10-fold decrease of activity on hDAT with respect to paroxetine.

### 3D-QSAR Modeling of Known Ligands

The best GOLD docking poses of compounds **1**–**19** were used as a training set alignment for constructing a 3D-QSAR model. The aim was to validate our transporter models through a quantitative description of known inhibitors activities. Unfortunately, the inhibition data of known inhibitors against SERT, DAT, and NET are usually related to different species, and only a small amount of information about human transporters collected in homologues assays is available. However, in a semiquantitative manner, it is possible to consider test results performed in rodent and human tissues as comparable since human and mouse transporters are similar in their sensitivities to tested drugs.^28^ Therefore, the 3D-QSAR could be considered a good method for model validations.

The collected activities of 19 known compounds were used to perform a Leave One Out (LOO) cross-validated partial least-squares (PLS) analysis on the GRID MIFs^32^ generated through the FLAP program^31^ on the relative docking poses. Their activities are shown in Table 2, in comparison with the ones predicted through the resulting 3D-QSAR models.

**Table 2:** Binding Affinity (p*K*_i_) of Known Compounds **1**–**19** for DAT, NET, and SERT^m^

	DAT p*K*_i_		NET p*K*_i_		SERT p*K*_i_	
compound	exp	pred	exp	pred	exp	pred
**1**: paroxetine^a^^,^^k^	6.31	5.86	7.4	7.03	10	8.80
**2**: fluoxetine^a^^,^^k^	5.42	5.5	6.62	5.59	9.1	7.85
**3**: escitalopram^b^^,^^k^	5.2	5.78	5	6.45	8.7	7.39
**4**: femoxetine^a^^,^^k^	5.7	6.52	6.12	6.29	7.96	7.76
**5**: esreboxetine^c^^,^^k^	5.2	5.2	8.98	8.29	6.18	6.47
**6**: atomoxetine^d^^,^^k^	5.8	6.1	8.30	8.59	7.14	6.88
**7**: maprotiline^a^^,^^k^	6	5.96	7.95	7.21	5.24	6.62
**8**: nisoxetine^e^^,^^l^	6.3	6	9.34	8.15	6.8	7.80
**9**: RTI-31^f^^,^^k^	8.57	8.1	7.4	7.70	7.7	8.18
**10**: RTI-55^f^^,^^k^	8.41	8.2	7.7	7.33	8.4	7.97
**11**: fluvoxamine^a^^,^^k^	5.03	6.09	5.89	6.29	8.7	8.97
**12**: GBR-12909^g^^,^^l^	7.92	7.02	5.9	6.16	6.98	6.96
**13**: GBR-12935^h^^,^^k^	7.14	7.19	6.2	6.44	5.7	5.88
**14**: indalpine^i^^,^^k^	6	6.22	6.29	7.10	8.76	8.46
**15**: sertraline^a^^,^^k^	7.60	6	6.38	6.99	9.52	8.56
**16**: viloxazine^a^^,^^k^	5	6.19	6.81	7.65	4.76	6.65
**17**: RTI-229^j^^,^^k^			6.22	6.16	6.8	7.19
**18**: zimelidine^a^^,^^k^	4.93	6.14	5.03	5.77	6.82	6.66
**19**: RTI-113^j^^,^^l^			5.75	6.04	6.67	6.86

a Reference (39).

b Reference (41).

c Reference (42).

d Reference (43).

e Reference (44).

f Reference (45).

g Reference (46).

h Reference (47).

i Reference (48).

j Reference (49).

k Binding affinity for human transporters.

l Binding affinity for rat transporter.

m For each transporter, the experimental and predicted p*K*_i_ values are reported.

In the 3D-QSAR models generated using the docking poses in hSERT, the first PLS component explained 83% of variance and was only quietly predictive (*Q*^2^ = 0.532), but the second PLS component improved the fitting (*R*^2^ = 0.966) and the predictive ability of the model (*Q*^2^ = 0.640). The third, fourth, and fifth PLS components provided further improvement in fitting (LV3: *R*^2^ = 0.994, *Q*^2^ = 0.657; LV4: *R*^2^ = 0.997, *Q*^2^ = 0.657; LV5: *R*^2^ = 0.999, *Q*^2^ = 0.660), whereas the sixth PLS component provided no further significant improvement. Thus, the model optimal dimensionality was given by five components. Also for hNET and hDAT models, the LV5 model showed the best 3D-QSAR capabilities with fitting and predictivity similar to the hSERT model (see Table 5). The experimental/predicted plots reported in Figure 8 for the training set (compounds **1**–**19** in blue) show a similar trend and a good predictivity for the three transporters. Unfortunately, for hDAT, the biological activity space is not distributed as for the other transporters. Almost all training set compounds cover a span of affinity in the 5–7 range of p*K*_i_ in hDAT. For the best inhibitors RTI-229 and RTI-113, only inhibition values of dopamine uptake in rats are known. Anyway, the predictivity range between 7 and 9 can be considered very good except for sertraline, and the good *R*^2^ and *Q*^2^ values provided a statistical validation of our transporter models.

The best GOLD docking poses of compounds **20a–d** and **21a–d** were used as an external test set for validating our 3D-QSAR models in a semiquantitative way even though our compounds were tested using rabbit cerebral tissue. Calculations produced predicted *K*_i_ values for all compounds in the millimolar and nanomolar ranges for hDAT and hSERT, respectively. Good predictions were obtained for compounds **20b**, **20d**, **21b**, and **21d**. Prediction values are reported in the next sections together with compound experimental results.

### Chemistry

**Scheme 1:**
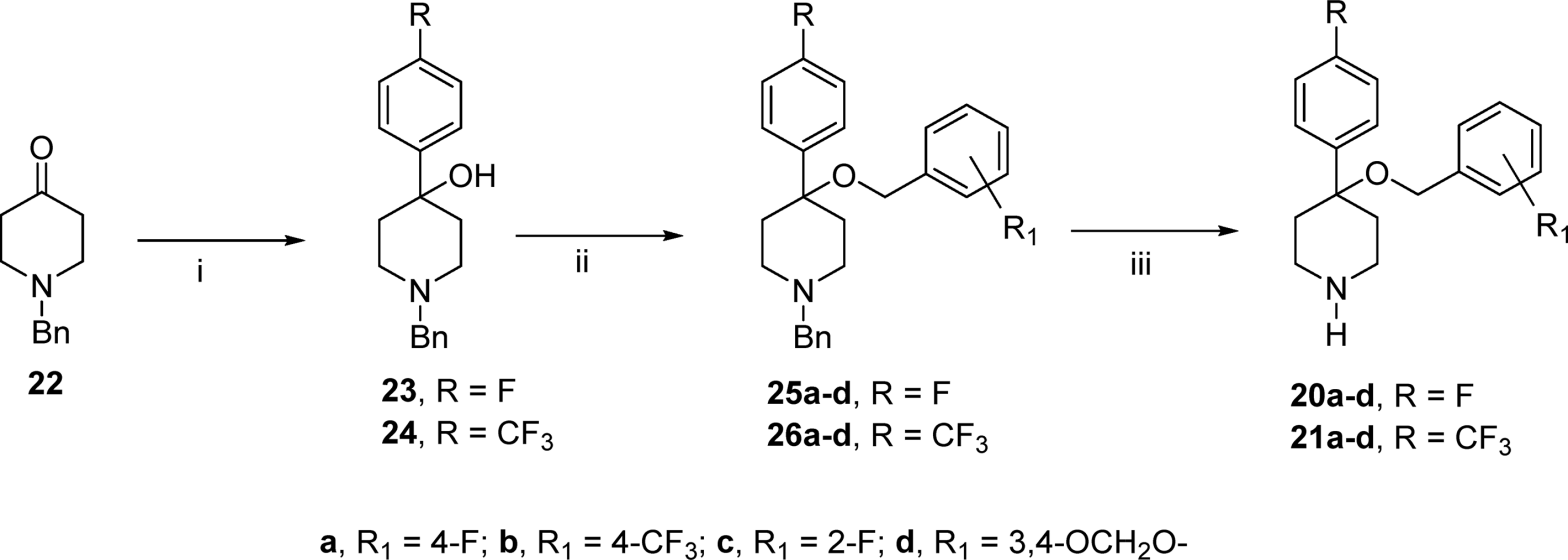


The synthesis of 4-((4-aryl)methyloxy)-4-(4-fluorophenyl)piperidine **20a**–**d** (R = F) and **21a**–**d** (R = CF_3_) is described in Scheme 1. 1-Benzyl-4-piperidone (**22**) was reacted with the appropriate aryl Grignard reagent in anhydrous tetrahydrofuran (THF) at reflux temperature to give the 4-piperidinols **23** and **24** in good yields. By reaction of the sodium salt of **23** or **24** dissolved in anhydrous THF with the appropriate benzyl bromide in the presence of tetrabutylamoniun iodide were obtained the ethers **25a**–**d** and **26a–d.** The catalytic hydrogenolysis of **25a**–**d** and **26a**–**d** in acidic medium gave the 4-(arylphenyl)-4-[(4-aryl)benzyloxy]piperidines **20a**–**d** and **21a**–**d** as hydrochloride salts.

### Biochemical Studies

#### Radioligand Binding Studies

The same experimental procedure already described for evaluating **20b**, **20d**, **21b**, and **21d** compound affinities towards SERT and DAT^21^ was used for **20a**, **20c**, **21a**, and **21c** compounds. Results (Table 3) showed that the R_1_ substitution with 2-F or 4-F produced a decrement in the affinity for SERT with respect to the bulkier CF_3_ in the 4 position or a piperonylic group. In order to complete their selectivity profile toward all transporters, all compounds were tested for the ability to displace [^3^H]nisoxetine binding to NET in rabbit cortical membrane. Overall our experimental data (Table 3) confirmed that **20b**, **20d**, **21b**, and **21d** compounds but also the new tested **20a, 20c, 21a**, and **21c** compounds possess a very high affinity toward SERT. In fact, all compounds are potent inhibitors of [^3^H]paroxetine binding to SERT showing *K*_i_ values within the nanomolar range, while the *K*_i_ values for displacing [^3^H]-WIN 35,428 binding to DAT are in the micromolar range (Table 3). Furthermore, the binding assays also revealed an affinity ratio SERT/NET higher than 10,000 for the best inhibitors, **20b**, **20d**, **21b**, and **21d**. In particular, **20b** and **20d** compounds seem to be 10-fold more potent inhibitors of [^3^H]paroxetine binding than unlabeled paroxetine and also show a higher selectivity toward SERT.

**Table 3:** Competition of **20a**–**d** and **21a**–**d** Compounds of [^3^H]-WIN 35,428 Binding to Rabbit Striatal Membranes, [^3^H]Nisoxetine, and [^3^H]Paroxetine Binding to Rabbit Cortical Membranes

compound^b^	[^3^H]-WIN 35,428, *K*_i_^a^ (nM)	[^3^H]nisoxetine, *K*_i_^a^ (nM)	[^3^H]paroxetine, *K*_i_^a^ (nM)
**20a**	>100,000	520	1.68 ± 0.49
**20b**	>100,000^c^	10,000	0.027 ± 0.005^c^
**20c**	>100,000	400	32.55 ± 12.56
**20d**	>100,000^c^	1,740	0.034 ± 0.0098^c^
**21a**	>100,000	>10,000	14.13 ± 3.04
**21b**	>100,000^c^	>10,000	0.316 ± 0.101^c^
**21c**	11,100 ± 4,900	2,070	50.02 ± 18.93
**21d**	11,200 ± 2,700^c^	2,450	0.250 ± 0.07^c^
paroxetine^d^	769^e^	80 ± 1	0.31 ± 0.018

a The *K*_i_ values are expressed as the mean ± SE of three or more independent experiments.

b Prepared and tested as hydrochloride salts.

c Reference (21).

d Reference (29).

e The *K*_i_ value represents the average of two independent experiments.

Stimulated by the 3D-QSAR results, the ability of the most active compounds (**20b**, **20d**, **21b**, and **21d**) to inhibit [^3^H]paroxetine binding to human platelet membranes was also investigated with the aim of assessing whether they displayed similar inhibition potencies toward rabbit and human SERT and verifying whether the underestimation in the 3D-QSAR predictions could be due to species differences. Indeed, the *K*_d_ value of paroxetine for rabbit SERT is 0.056 nM,^29^ while the *K*_d_ value for hSERT reported in the literature is 0.1 nM.^40^ The *K*_i_ values of tested compounds and paroxetine for inhibiting [^3^H]paroxetine binding to human platelet membranes are shown in Table 4. The results show that the ability of synthesized compounds to inhibit [^3^H]paroxetine binding to SERT in human platelet membranes was in the low nanomolar range with a subnanomolar *K*_i_ value (0.08 nM) for **20b**. These values are of 1 order of magnitude higher than the ones measured in rabbit tissue.

**Table 4:** Inhibition Constants (*K*_i_) of **20a, 20c, 21a**, and **21c** Compounds and Paroxetine for Inhibiting [^3^H]Paroxetine Binding and the [^3^H]-5-HT Uptake to SERT in Human Platelets

	binding inhibition	[^3^H]-5-HT uptake inhibition	
compound	[3H]paroxetine *K*_i_^a^ (nM)	IC_50_ (nM)^b^	*K*_i_^a^ (nM)^c^
**20b**	0.17 ± 0.02	0.13 ± 0.01	0.08
**20d**	1.71 ± 0.15	2.64 ± 0.3	1.75
**21b**	5.60 ± 0.52	4.20 ± 0.4	2.77
**21d**	8.72 ± 0.93	4.90 ± 0.5	3.26
paroxetine	0.09 ± 0.01	0.11 ± 0.01	0.051

a The *K*_i_ values are expressed as the mean ± SE of three or more independent experiments.

b [^3^H]-5-HT (25 nM) and increasing concentrations (0.01–1000 nM) of compounds or paroxetine were used.

c The *K*_i_ values were derived from *K*_m_ values determined using a fixed concentration of compounds or paroxetine in saturation experiments of the [^3^H]-5-HT uptake as described in the Methods section.

#### Functional Studies

##### SERT Uptake Experiment

The uptake inhibitory activities of compounds (**20b**, **20d**, **21b**, and **21d**) were measured by [^3^H]5-HT uptake kinetic experiments on human platelets. The *K*_m_ value for the [^3^H]5-HT uptake was determined in saturating conditions as described in the Methods section. Under these conditions, the [^3^H]5-HT uptake showed a *K*_m_ of 87.59 nM and a *V*_max_ of 132.7 pmol/10^9^ plt/min. The Michaelis–Menten constant for substrate was determined from the initial rate measurements at 37 °C by a nonlinear regression analysis using the GraphPad Prism 5.0 program.

**Figure 7:**
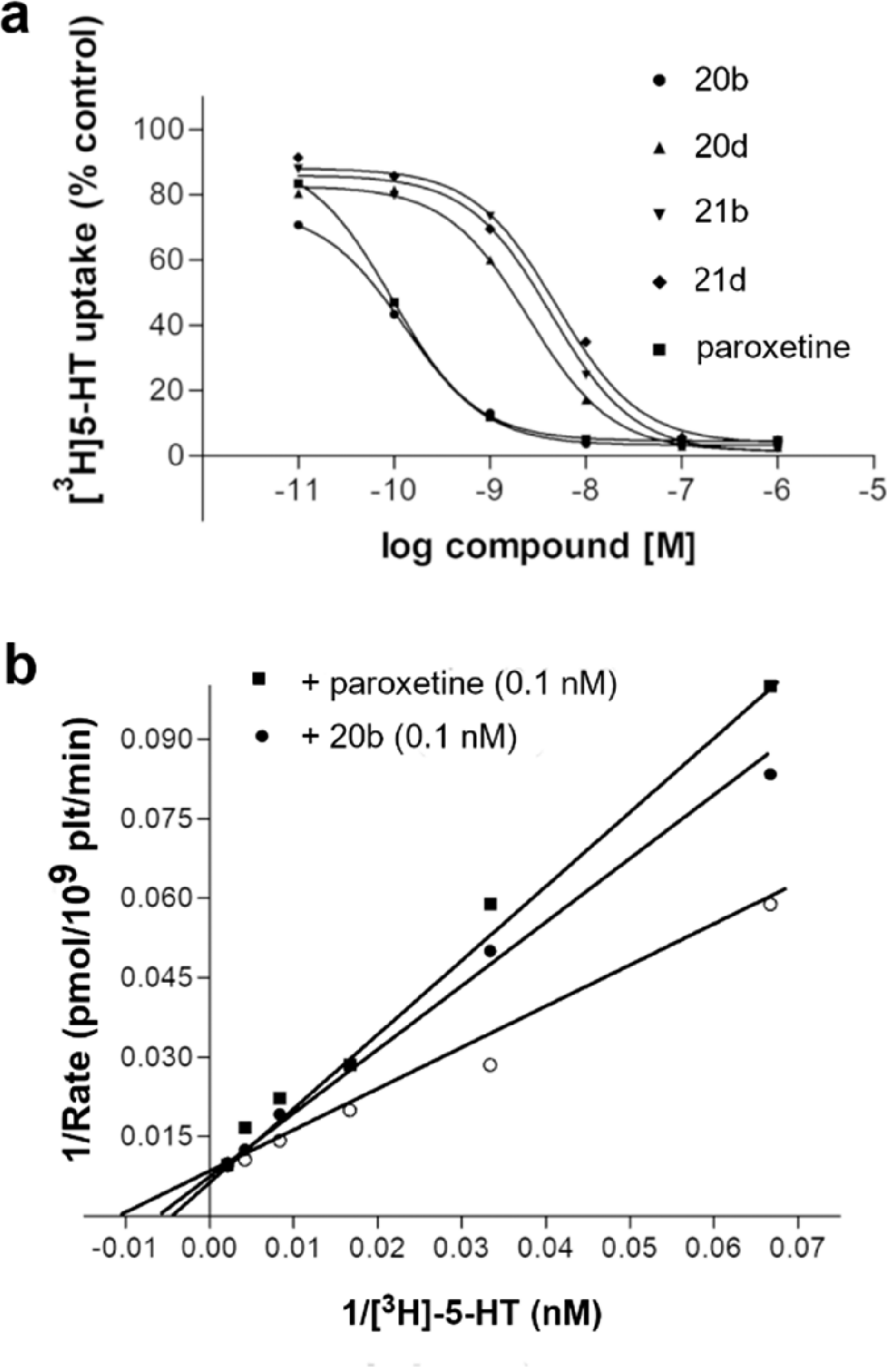
a) Inhibition of the [^3^H]5-HT uptake in human platelets by **20b**, **20d**, **21b**, and **21d** compounds. Platelets were incubated in duplicate with [^3^H]5-HT in the presence and absence of increasing concentrations of each compound as described in the Methods section. b) The Lineweaver–Burk plot showing competitive inhibition of the [^3^H]5-HT uptake by compound **20b** and paroxetine.

In order to study compound activity, we initially verified whether they caused a 50% inhibition of the specific [^3^H]5-HT uptake, and thus the IC_50_ (inhibition constant at 50% of control) values could be determined. The inhibitory activity of compounds and paroxetine was assessed using 25 nM [^3^H]5-HT and six different concentrations of the inhibitors. The inhibition curves are shown in Figure 7 (panel a), while the derived IC_50_ values are reported in Table 4.

Compounds were further characterized by means of full uptake kinetics, to verify the type of inhibition. Hence, saturation experiments of the [^3^H]5-HT uptake were performed in the presence and absence of compounds (0.1 nM) or paroxetine (0.1 nM) using six different [^3^H]5-HT concentrations (10 to 1,000 nM). Thus, the apparent *K*_m_ and *V*_max_ values of the [^3^H]5-HT uptake in the presence of inhibitors were determined using the Lineweaver–Burk plot. The *K*_i_ values (Table 4) of each compound and paroxetine were derived using the apparent *K*_m_ and the *K*_m_ value obtained in the absence of the inhibitor.

The competitive behavior of the most potent inhibitor (**20b**) is demonstrated by the fact that different concentrations of the compound did not modify the *V*_max_ value of the [^3^H]5-HT uptake kinetic, while the *K*_m_ value changed. In Figure 7 (panel b), the competitive behavior of compound **20b** and paroxetine for the [^3^H]5-HT uptake is graphically shown by the Lineweaver–Burk plot. To mention, the *K*_i_ value of such a compound, which is within the low nanomolar range (*K*_i_, 0.08 nM), is similar to the one of paroxetine (*K*_i_, 0.05 nM (Table 4)).

### 3D-QSAR Prediction of **20a–d** and **21a–d** Compounds

**Figure 8:**
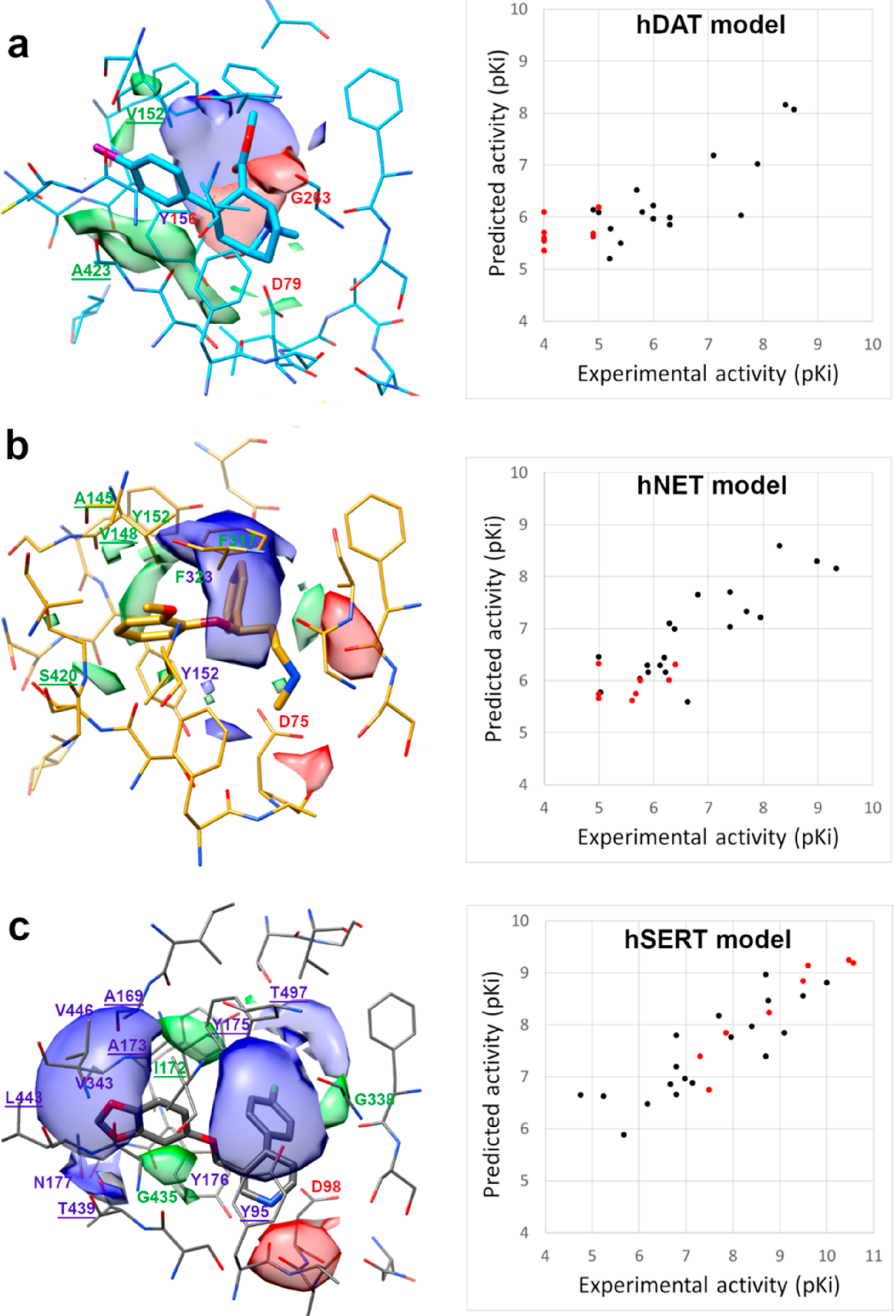
Contour maps of the PLS pseudocoefficient plots obtained with the N1 (blue), O (red), and DRY (green) probes superposed on hDAT-RTI-55 (a), hNET-nisoxetine (b), and hSERT-paroxetine (c) models. Energy levels are set at −0.206 (range −0.636–0) for N1 MIF, −1.17 (range −3.01–0) for DRY MIF, and −0.603 (range −1.63–0) for O MIF in a), −0.852 (range −2.39–0) for N1 MIF, −0.268 (range −0.807–0) for DRY MIF, and −0.773 (range −2.22–0) for O MIF in b), and −0.789 (range −2.05–0) for N1 MIF, −0.196 (range −0.603–0) for DRY MIF, and −0.552 (range −1.46–0) for O MIF in c). On the left, plot of the predicted against experimental activity of a training set (black) and an external test set (red) against hDAT (a), hNET (b), and hSERT (c).

The best GOLD docking poses of **20a**–**d** and **21a**–**d** compounds were used as an external test set for our 3D-QSAR models. Results in terms of SDEP*ext* are reported in Table 5, while the predicted activities over the experimental ones are plotted in Figure 8. Unfortunately, the experimental results on DAT reported in Table 3 were not adequate for an external prediction since the *K*_i_ values of these compounds were not in the same applicability domain of our hDAT 3D-QSAR model.

**Table 5:** Statistical Results of the 3D-QSAR Calculation

DAT	NET	SERT
*R*^2^ 0.999	*R*^2^ 0.997	*R*^2^ 0.999
*Q*^2^ 0.62	*Q*^2^ 0.68	*Q*^2^ 0.66
(SDEP*ext* 1.46)	SDEP*ext* 0.60	SDEP*ext* 0.78

The SDEP*ext* values are satisfactory for hNET and hSERT, whereas the SDEP*ext* value is unreliable for hDAT.

In the hSERT model, the external test set covered a wide range of activities, so we tried to use this model to study the species dependence of the inhibition results. A first attempt was the construction of the hSERT 3D-QSAR model using only compounds tested on human transporter and subsequent prediction of the rat experimental data as an external test set. The result in terms of SDEP was 0.6, comparable to the *Q*^2^ value relative to our model (Table 5) generated using human and rat data as the training set. This result seems to confirm a similar sensitivity of human and mouse transporters to tested drugs. On the contrary, the results produced in rabbit SERT and predicted using our hSERT model showed an SDEP*ext* value of 0.78. As shown in Figure S7, this higher value than the one of *Q*^2^ is due to an underestimation of almost all predicted inhibition values. This result seems to suggest a different sensitivity of rabbit SERT compared with the human one toward our compounds. Therefore, the most potent compounds (**20a**, **20c**, **21a**, and **21c)** were experimentally tested for inhibiting [^3^H]paroxetine binding to SERT in human platelet membranes (Table 4). The insertion of the new *K*_i_ values in our 3D-QSAR model led to a decrease of the extSDEP to 0.65. At this point, the *K*_i_ values predicted by our hSERT model were of the same extent of those measured using human platelet membranes. Thus, a discrepancy of about 1 order of magnitude of compound activities toward the rabbit- and human SERT was validated. Nevertheless, the trend of the inhibitory potencies is conserved between the two species.

The MIFs generated during the 3D-QSAR analysis on the docking poses could be represented in a graphical mode using the PLS pseudocoefficient plots.^50^ They are very useful to visualize favorable interactions between the DRY, N1, and O probes and the molecules studied. Such regions are related to the ligand poses and are independent from the protein during their generation. The superposition of these maps with transporter could give information about the regions of the protein, which enhance the activity and are responsible for the selectivity. Figure 8a–c represents the comparison of the PLS pseudocoefficient contour plots for DRY (green polyhedrons), N1 (blue polyhedrons), and O (red polyhedrons) probes overlapping the hDAT-RTI-55 (a), hNET-nisoxetine (b), and hSERT-paroxetine (c) complexes. All maps are visualized at the same relative energy value. It is immediately clear the importance of the donor probe in hSERT and the DRY probe in hDAT, but a more deep analysis of the interactions is required for MIFs interpretation.

For all transporters the lipophilic contribution of the not conserved S149/A145/A169, V152/V148/I172, and A423/S420/T439 residues is decisive although different for hDAT, hNET, and hSERT. These mutations, already highlighted in another study,^51^ are responsible for the different stabilizations of one of the inhibitor aromatic moieties and ring substitution effects on biological activity and transporter selectivity. In particular, Ala423 in place of a Ser or Thr residue in hNET and hSERT enhances the favorable lipophilic region in hDAT (green in Figure 8b) with respect to the other transporters. In hDAT, a large blue region, which is favorable for a donor probe, is superposed and fused with a red one (favorable for an acceptor probe). This map seems to be due to a moiety capable of being both donor and acceptor. Flexibility of Tyr156 could be responsible for the blue-red region through its OH group. In hNET, a quite large blue region related to the N1 probe is perfectly overlapped to the Phe272 position. This discordance could be solved by checking in detail the behavior of MIFs at different energy values (Figure S6) in the Phe323 region. The PLS pseudocoefficient plot of the DRY probe at a higher energy level (points of medium interaction with a lipophilic probe) overlaps the N1 probe plot at a lower energy level (points of maximum interaction with a donor probe). This mixed lipophilic-donor region corresponded to the ethers of nisoxetine, esreboxetine, and atomoxetine docking poses, for which FLAP calculated a top stabilizing effect by an electron rich donor probe and a moderate stabilizing effect by a lipophilic group. A Phe residue cannot engage hydrogen bonds but can form oxygen lp−π interactions.

Human SERT showed the bigger blue area (Figure 8c) formed by four regions as follows: the first corresponding to Thr439, the second to Tyr95, and the third to Thr497, which are not conserved residues. The latest blue region seems to be incoherent with the overlapping protein residues. This N1 MIF corresponds to Ala169, Ala173, Val343, and Leu443, which are all lipophilic residues. In this case, FLAP did not calculate any favorable interaction with the DRY probe in this region, which is related to halogens of fluvoxamine, sertraline, and fluoxetine. This is the real “halogen binding pocket” highlighted by crystallographic structures.

In all transporters, limited red regions related to the O probe are just calculated near the flexible conserved Asp, whose interaction with the inhibitor amine is mandatory for the activity.

In summary, FLAP MIFs calculated on the docking pose of inhibitors after superposition on the protein structure were able to detect some unconserved residues as key elements for transporter selectivity. Small mutations spreading to the binding site, such as F76/F72/Y95, S149/A145/A169, V152/V148/I172, G153/G149/A173, F155/Y151/Y175, A423/S420/T439, and A480/A477/T497, influence the cavities shaping and polarity and allow a different stabilization of the inhibitors in the three transporters. These mutations seem to be statistically correlated to the chemical features of classical inhibitors, whose docking in hDAT, hNET, and hSERT models also revealed the importance of the cavity delimited by Trp84, Arg85, Asp476, Pro387, and Phe472 for the hDAT selectivity. In particular, the role of the unconserved Asp476 and Phe472 seems to be essential in stabilizing “atypical” DAT inhibitors. In hSERT, a pocket widening in the zone labeled as S-SERT near Phe431 (Figure 3c) which is influenced by the conserved Ile172 seems to be involved in the stabilization of selective hSERT inhibitors. The three-dimensional model of the three human transporters aided to rationalize the activities of **20a**–**d** and **21a**–**d** compounds, which are able to strongly inhibit [^3^H]paroxetine binding to SERT in rabbit membranes and also show a quite discriminative power between SERT and the other transporters. Although the most active compounds showed 1 order of magnitude lower potency in human platelet membranes than in rabbit cortical membranes, all of the data point out that the **20b** compound possesses a very interesting profile and our three-dimensional and 3D-QSAR models can represent promising tools for predicting the inhibitory activity of new molecules targeting human transporters.

## Methods

### Chemistry

Analytical grade reagents and solvent were purchased from Sigma-Aldrich (St. Louis, MO) and were used as supplied. Solvents were dried according to standard methods. Melting points were determined on a Köfler hot-stage apparatus and are uncorrected. ^1^H NMR spectra were recorded with a Varian Gemini-200 MHz spectrometer in an ca. 2% solution of CDCl_3_. Chemical shifts (δ) are reported in parts per million (ppm) downfield from tetramethylsilane (TMS) as internal standard. The following abbreviations are used: singlet (s), broad (br), and multiplet (m). Reactions were monitored by thin layer chromatography (TLC) on silica gel plates containing a fluorescent indicator (Merck Silica Gel 60 F254), and spots were detected under UV light (254 nm). Chromatographic separations were performed on silica gel columns by flash column chromatography (Kieselgel 40, 0.040–0.063 mm; Merck). Na_2_SO_4_ was always used as the drying agent. Evaporation was carried out “in vacuo” (rotating evaporator). Elemental analyses were performed by our analytical laboratory and agreed with the theoretical values to within ±0.4%.

#### Synthesis of 1-Benzyl-4-(4-fluorophenyl)piperidin-4-ol (**4**) and 1-Benzyl-4-(4-(trifluoromethyl)phenyl)piperidin-4-ol (**5**)

These compounds were prepared slightly modifying the synthetic route previously described.^29^ In brief: the opportune 4-(aryl)magnesium bromide prepared in the usual manner and refluxed under stirring for 30 min was treated dropwise at room temperature with a THF solution of *N*-benzyl-4-piperidone (**3**), and then the solution was refluxed under stirring for 15 h. After the usual workup, the oily residue so obtained was crystallized from hexane to give pure **4** as a pale yellow solid or purified by column chromatography on silica gel (EtOAc/hexane 4:6) to give pure **5** as a white solid.

#### General Procedure for the Synthesis of 1-Benzyl-4-aryloxy-4-arylpiperidine **25a**–**d** and **26a**–**d**

To a stirred solution of the opportune 1-benzyl-4-(4-fluorophenyl)piperidin-4-ol **4** or 1-benzyl-4-(4-(trifluoromethyl)phenyl)piperidin-4-ol **5** (1.76 mmol) in anhydrous THF (10 mL) was added NaH 60% (1.84 mmol) under nitrogen atmosphere. Then to the reaction mixture was added tetrabutylammonium bromide (0.018 mmol) and, dropwise under stirring, a solution of the opportune benzyl chloride (0.78 mmol). The mixture was stirred for 3 days at room temperature and then water was added, and the solution was extracted with EtOAc. The organic extracts were evaporated to yield a crude oil which was purified by column chromatography on silica gel (EtOAc/hexane 4:6) to yield the compounds **25a**–**d** or **26a**–**d**.

##### 1-Benzyl-4-((4-fluorobenzyl)oxy)-4-(4-fluorophenyl)piperidine (**25a**) Characterization

(45%) ^1^H NMR (200 MHz, CDCl_3_): δ 7.46–6.97 (m, 13H), 4.03 (s, 2H), 3.58 (s, 2H), 2.77 (m, 2H), 2.51 (m, 2H), 2.10 (m, 4H). Anal. Calcd for C_25_H_25_F_2_NO: C 76.31; H 6.40; N 3.56; found: C 76.45; H 6.27; N 3.71.

##### 1-Benzyl-4-(4-fluorophenyl)-4-((4-(trifluoromethyl)benzyl)oxy)piperidine (**25b**) Characterization

(50%) ^1^H NMR (200 MHz, CDCl_3_): δ 7.46–6.97 (m, 13H), 4.03 (s, 2H), 3.62 (s, 2H), 2.82 (m, 2H), 2.57 (m, 2H), 2.13 (m, 4H). Anal. Calcd for C_26_H_25_F_4_NO: C 70.42; H 5.68; N 3.16; found: C 70.21; H 5.74; N 3.05.

##### 1-Benzyl-4-((2-fluorobenzyl)oxy)-4-(4-fluorophenyl)piperidine (**25c**) Characterization

(85%) ^1^H NMR (200 MHz, CDCl_3_): δ 7.59–7.01 (m, 13H), 4.15 (s, 2H), 3.58 (s, 2H), 2.79 (m, 2H), 2.53 (m, 2H), 2.10 (m, 4H). Anal. Calcd for C_25_H_25_F_2_NO C 76.31; H 6.40; N 3.56; found: C 76.25; H 6.22; N 3.73.

##### 4-(Benzo[D][1,3]dioxol-5-ylmethoxy)-1-benzyl-4-(4-fluorophenyl)piperidine (**25d**) Characterization

(59%) ^1^H NMR (200 MHz, CDCl_3_): δ 7.46–6.67 (m, 12H), 5.95 (s, 2H), 3.96 (s, 2H), 3.57 (s, 2H), 2.75 (m, 2H), 2.52 (m, 2H), 2.08 (m, 4H). Anal. Calcd for. C_26_H_26_FNO_3_ C 74.44; H 6.25; N, 3.34; found: C 74.31; H 6.14; N 3.23.

##### 1-Benzyl-4-((4-fluorobenzyl)oxy)-4-(4-(trifluoromethyl)phenyl)piperidine (**26a**) Characterization

(85%) ^1^H NMR (200 MHz, CDCl_3_): δ 7.65–6.97 (m, 13H), 4.05 (s, 2H), 3.62 (s, 2H), 2.82 (m, 2H), 2.58 (m, 2H), 2.13 (m, 4H). Anal. Calcd for C_26_H_25_F_4_NO: C 70.42; H 5.68; N 3.16; found: C 70.36; H 5.59; N 3.21.

##### 1-Benzyl-4-((4-(trifluoromethyl)benzyl)oxy)-4-(4-(trifluoromethyl)phenyl)piperidine (**26b**) Characterization

(70%) ^1^H NMR (200 MHz, CDCl_3_): δ 7.66–7.01 (m, 13H), 4.12 (s, 2H), 3.57 (s, 2H), 2.77 (m, 2H), 2.51 (m, 2H), 2.13 (m, 4H). Anal. Calcd for C_27_H_25_F_6_NO: C, 65.71; H, 5.11; N, 2.84; found: C, 65.52; H, 5.20; N, 2.76.

##### 1-Benzyl-4-((2-fluorobenzyl)oxy)-4-(4-(trifluoromethyl)phenyl)piperidine (**26c**) Characterization

(85%) ^1^H NMR (200 MHz, CDCl_3_): δ 7.66–6.98 (m, 13H), 4.17 (s, 2H), 3.58 (s, 2H), 2.79 (m, 2H), 2.52 (m, 2H), 2.14 (m, 4H). Anal. Calcd for C_26_H_25_F_4_NO: C 70.42; H 5.68; N 3.16; found: C 70.61; H 5.42; N 3.21.

##### 4-(Benzo[D][1,3]dioxol-5-ylmethoxy)-1-benzyl-4-(4-(trifluoromethyl)phenyl)piperidine (**26d**) Characterization

(50%) ^1^H NMR (200 MHz, CDCl_3_): δ 7.47–6.67 (m, 12H), 5.95 (s, 2H), 3.95 (s, 2H), 3.56 (s, 2H), 2.75 (m, 2H), 2.51 (m, 2H), 2.08 (m, 4H). Anal. Calcd for C_27_H_26_F_3_NO: C, 69.07; H, 5.58; N, 2.98; found: C, 68.92; H, 5.65; N, 2.87.

##### General Procedure for the Synthesis of 4-Aryloxy-4-arylpiperidine Hydrochlorides **20a–d** and **21a–d**

To a solution of **25a**–**d** and **26a**–**d** (0.82 mmol) in EtOH anhydrous (50 mL) was added a solution of EtOH·HCl to pH ≈ 3. The mixture was shaken under hydrogen at room temperature and atmospheric pressure for 24 h in the presence of 10% Pd on charcoal (65 mg), then the catalyst was filtered off, and the solution was evaporated to yield the crude piperidine hydrochlorides that were crystallized by Et_2_O to give **20a**–**d** and **21a–d.**

##### 4-((4-Fluorobenzyl)oxy)-4-(4-fluorophenyl)piperidine Hydrochloride (**20a**) Characterization

(80%) mp 158–159 °C; ^1^H NMR: (200 MHz, CDCl_3_): δ 9.59 (brs, 1H), 7.39–6.99 (m, 8H), 4.03 (s, 2H), 3.42 (m, 4H), 2.36 (m, 4H). Anal. Calcd for C_18_H_20_ClF_2_NO: C, 63.62; H, 5.93; N, 4.12; found: C, 63.52; H, 5.99; N, 4.03.

##### 4-(4-Fluorophenyl)-4-((4-(trifluoromethyl)benzyl)oxy)piperidine Hydrochloride (**20b**) Characterization

(95%) mp 184–185 °C; ^1^H NMR (200 MHz, CDCl_3_): δ 9.64 (brs, 1H), 7.63–7.05 (m, 8H), 4.14 (s, 2H), 3.46 (m, 4H), 2.38 (m, 4H). Anal. Calcd for C_19_H_20_ClF_4_NO: C, 58.54; H, 5.17; N, 3.59; found: C, 58.37; H, 5.22; N, 3.65.

##### 4-((2-Fluorobenzyl)oxy)-4-(4-fluorophenyl)piperidine Hydrochloride (**20c**) Characterization

(80%) mp 202 °C; ^1^H NMR (200 MHz, CDCl_3_): δ 7.43–7.00 (m, 8H), 4.11 (s, 2H), 3.45 (m, 4H), 2.39 (m, 4H). Anal. Calcd for C_18_H_20_ClF_2_NO: C, 63.62; H, 5.93; N, 4.12; found: C 63.75; H 5.87; N 4.23. MeOH/Et_2_O.

##### 4-(Benzo[D][1,3]dioxol-5-ylmethoxy)-4-(4-fluorophenyl)piperidine Hydrochloride (**20d**) Characterization

(70%) mp 202 °C; ^1^H NMR (200 MHz, CDCl_3_): δ 9.61 (brs, 1H), 7.43–6.66 (m, 7H), 5.97 (s, 2H), 3.97 (s, 2H), 3.44 (m, 4H), 2.35 (m, 4H). Anal. Calcd for C_19_H_220b_lFNO_3_: C, 62.38; H, 5.79; N, 3.83; found: C 62.45; H 5.82; N 3.68.

##### 4-((4-Fluorobenzyl)oxy)-4-(4-(trifluoromethyl)phenyl)piperidine Hydrochloride (**21a**) Characterization

(80%) mp 261 °C dec.; ^1^H NMR: (200 MHz, CDCl_3_): δ 9.74 (brs, 1H), 7.70–7.01 (m, 8H), 4.06 (s, 2H), 3.43 (m, 4H), 2.35 (m, 4H). Anal. Calcd for C_19_H_20_ClF_4_NO: C, 58.54; H, 5.17; N, 3.59; found: C 58.31; H, 5.23; N, 3.69.

##### 4-((4-(Trifluoromethyl)benzyl)oxy)-4-(4-(trifluoromethyl)phenyl)piperidine Hydrochloride (**21b**) Characterization

(65%) mp 189–190 °C; ^1^H NMR (200 MHz, CDCl_3_): δ 9.74 (brs, 1H), 7.70–7.36 (m, 8H), 4.17 (s, 2H), 3.37 (m, 4H), 2.38 (m, 4H). Anal. Calcd for C_20_H_20_ClF_6_NO: C, 54.62; H, 4.58; N, 3.18; found: C, 54.48; H, 4.42; N, 3.11.

##### 4-((2-Fluorobenzyl)oxy)-4-(4-(trifluoromethyl)phenyl)piperidine Hydrochloride (**21c**) Characterization

(70%) mp 210 °C dec.; ^1^H NMR (200 MHz, CDCl_3_): δ 9.74 (brs, 1H), 7.60–6.97 (m, 8H), 4.06 (s, 2H), 3.36 (m, 4H), 2.37 (m, 4H). Anal. Calcd for C_19_H_20_ClF_4_NO: C, 58.54; H, 5.17; N, 3.59; found: C 58.36; H, 5.11; N, 3.66.

##### 4-(Benzo[d][1,3]dioxol-5-ylmethoxy)-4-(4-(trifluoromethyl)phenyl)piperidine Hydrochloride (**21d**) Characterization

(70%) mp 231–233 °C; ^1^H NMR (200 MHz, CDCl_3_): δ 9.70 (brs, 1H), 7.71–6.98 (m, 7H), 5.99 (s, 2H), 4.01 (s, 2H), 3.45 (m, 4H), 2.35 (m, 4H). Anal. Calcd for C_20_H_220b_lF_3_NO_3_: C, 57.77; H, 5.09; N, 3.37; found: C, 57.89; H, 5.21; N, 3.24.

### Computational Studies

#### Human DAT and NET Modeling

The primary sequences of the transporters were retrieved from the UNIPROT protein sequence database (Q01959 and P23975, respectively).^24^ A BLAST^23^ search of these sequences against PDB^19^ sequence entries was performed. The BLAST-derived scores suggested a close homology between both transporters and the crystallized *Drosophila melanogaster* dopamine transporter (best scored the one complexed with cocaine, PDB code 4XP4)^52^ and with the hSERT-paroxetine crystallized complex (PDB code 5I6X).^53^ Therefore, their 3D coordinates were retrieved. A multiple structure alignment of all the human transporters on *Drosophila melanogaster* DAT was performed using Praline^54^ with a gap open penalty of 15 and a gap extension penalty of 1. For all the extra or intracellular loops, we used the crystal structures as a template due to the good quality of alignment. Only for the long EL2 loop of human transporters, namely for NET, the template is not so good for a 13 amino acid region, which lacks in the crystallized DAT. This region is shorter in the SERT structure where the gap is six residues long. The unaligned area is comprised between two beta-sheet regions in the extreme external zone at more than 26 Å from the binding site. Considering the area is unessential for the goal of this study, we decided to leave it free of template and assign the conformation of this short sequence through the simulated annealing.

The 3D models of hDAT and hNET were constructed using the MODELLER program^25^ on the basis of the alignment obtained from Praline. Cocaine was manually included in the binding sites of hDAT and hNET in the same conformation and orientation of the template crystal structures. MD simulations for 10 ns were performed for hDAT-cocaine and hNET-cocaine complexes embedded in DOPC bilayers. The CHARMM-GUI web server^55^ was employed in order to obtain a pre-equilibrated membrane. This was composed of 130 lipids embedded in a 80 × 80 Å^2^ square membrane, and the ligand-transporter complex was placed into the membrane orienting it along the *z-*axis. This structure was solvated with TIP3P water and 0.15 M KCl extending 15 Å at the top and bottom of the membrane. The system was rebuilt in Amber14^56^ using xLeap in order to generate a topology file, protonation, angles, and dihedrals. N- and C-termini of the protein model systems were capped by acetyl and methylamino groups. General Amber force field (GAFF) parameters were assigned to cocaine, while partial charges were calculated using the AM1-BCC method as implemented in the Antechamber suite of AMBER 14. The total number of atoms of each complex was approximately 69,000. The default particle mesh Ewald method (PME) was employed to calculate long-range electrostatic interactions with an Ewald coefficient of 0.275 Å. Van der Waals and short-range electrostatic interactions were smoothly truncated at 10.0 Å. The Langevin thermostat was utilized to equilibrate the temperature, and the anisotropic Berendsen barostat was used to control the pressure. Periodic boundary conditions were applied (80 × 80 × 120) Å.^3^ Ten thousand steps of steepest descent and conjugate gradient minimization were performed, with harmonic restraints of 50 kcal mol Å^–2^ applied on all solute atoms followed by 10,000 steps of minimization without restraints. The heating simulation was run in two phases: at first, a 200 ps simulation kept the system at 100 K in the NVT (constant number of particles, volume, and temperature) ensemble with protein complexes restrained with a force constant of 50 kcal mol Å^–2^, while lipids and ions were initially restrained with a force constant of 50 kcal mol Å^–2^ and then progressively relaxed (lipids after 80 ps, ions after 140 ps); then, the temperature was raised during a further 200 ps MD simulation to 300 K in the NPT ensemble with the same restraining scheme of the first heating. The temperature of 300 K was used in equilibration MD in order to ensure that the membrane state was well above the melting point of DOPC. An equilibration of 10 ns was performed in two stages: in the first 1 ns, the protein complexes were restrained with a force constant of 10 kcal mol Å^–2^ initially (400 ps) on all complex atoms and then only on the C alpha. The second stage of 9 ns was a NPT simulation without restraints.

The stereochemical quality of the resulting protein structures was evaluated by inspection of the Psi/Phi Ramachandran plot obtained from PROCHECK analysis.^27^ The MD snapshots were obtained through the MD/Ensamble Analysis module of Chimera.^57^

#### Optimization of wt-hSERT in Complex with Paroxetine

The wt-hSERT structure was obtained through Ala291Ile, Ser439Thr, Ala554Cys, and Ala580Cys mutations performed by Maestro in the 5I6X(53) PDB structure. The complex was embedded in a DOPC membrane using the already described procedure, and the system was subjected to the same simulation protocol of hNET and hDAT complexes.

#### Docking Procedure

Automated docking of the ligands into models was carried out by means of the GOLD 5.1 program^30^ and by Flapdock^31^ for a double check of the training set docking poses. The ligands were built using the Maestro program^58^ and subjected to a Conformational Search (CS) of 1,000 steps in a water environment using the Macromodel program.^58^ The Monte Carlo algorithm was used with the MMFFs force field. The ligands were then minimized using the Conjugated Gradient method to a convergence value of 0.05 kcal/Å·mol using the same force field and parameters as for the CS.

The region of interest was defined in GOLD^30^ in such a manner that it contains all residues within 10 Å from ligands. The “allow early termination” command was deactivated. All ligands were submitted to 40 Genetic Algorithm runs using the ChemScore fitness function, rescoring through the PLP function, and clustering the output orientations on the basis of a RMSD distance of 1.5 Å. The default GOLD parameters were used for all variables except for the side chains rotamers. The EXTRA PARAMETER option was used to allow the free side chain flexibility of Asp79 and Phe320, Asp75 and Phe317, and Asp98 and Phe335 for hDAT, hNET, and hSERT, respectively. The best docking pose for each ligand was then used for further studies.

The FLAP database of ligands was generated using the standard GRID probes H, DRY, N1, and O with a spatial resolution of 0.75 Å. For each ligand, up to 25 conformers were generated with an RMSD cutoff of 0.3 Å between two conformers. The H probe describes the shape of the molecule, whereas the DRY probe detects hydrophobic interactions. The hydrogen-bond acceptor and hydrogen-bond donor capacities of the target are described by the amide N1 and carbonyl O probe, respectively. Transporter proteins were loaded specifying Na^+^ and Cl^–^ as metals, while RTI-55, nisoxetine, and paroxetine were imported as reference ligands for hDAT, hNET, and hSERT, respectively. The region of interest was defined within 10 Å from ligands. All default parameters were used for docking, and the best five poses ranked by S-Score were analyzed. The self-docking of RTI-55, nisoxetine, and paroxetine produced good results ranking the five best poses on the basis of H*DRY scores (shape combined with hydrophobicity). In this way, the contribution of small mutations spreading on the pocket shape and lipophilic stabilization of ligands was particularly taken into account. This procedure gave a self-docking result calculated as mean RMSD between predicted and reference poses of 0.8 Å. So, the best H*DRY scored poses for all ligands were compared with the GOLD results in terms of RMSD. The graphical analysis of the docking results was performed by Chimera.^57^

#### 3D-QSAR Modeling

The docking conformations of known SERT, DAT, and NET inhibitors were used as transporter-based alignments to construct a FLAP^31^ database for each transporter. FLAP (Fingerprints for Ligands And Proteins) is able to compare molecules using fingerprints. The fingerprints are derived from the GRID Molecular Interaction Fields (MIFs) and/or the GRID atom types and are characterized as quadruplets of pharmacophoric features. The MIFs produced by the GRID force field describe the type, strength, and direction of the interactions owed to a molecule. A quantitative examination of the MIF contributions to the activity of a set of aligned structures allows the construction of 3D-QSAR models. In this context, the GOLD docking conformer of each ligand was imported in the FLAP database. MIFs were then calculated using the acceptor (O), donor (N1), hydrophobic (DRY), and shape (H) probes as implemented in FLAP and using a grid resolution of 0.75 Å.

The interaction point energies were defined as independent variables, while inhibitor activity expressed as p*K*_i_ was set as the dependent variable. So, the docked data set was used as the training set to construct 3D-QSAR models analyzing through PLS the combinations of descriptors, which best explain the activity. The models were cross-validated using the LOO method and analyzed in terms of *R*^2^ and *Q*^2^.

The optimal number of latent variables was chosen for each model, and the prediction capability of the models toward each inhibitor was examined. To perform a study on selectivity, the MIF coefficients of each transporter model were plotted as isocontours comparing in a 3D view the most relevant MIFs, which represent the regions of a favorable interaction between an inhibitor substituent and the probes resulting in an increase of activity with hSERT, hDAT, and hNET binding site regions.

### Radioligand Binding and Functional Studies

#### Membrane Preparation for Radioligand Binding Studies

Cerebral tissue was from adult New Zealand White rabbits (4–5 kg) obtained from a commercial source (Charles River Laboratories, Inc., Wilmington, MA). Animals were maintained in standard laboratory conditions and fed in sawdust-lined cages and at a 12-h light/dark cycle. They were killed by intravenous injection of a lethal dose of pentobarbital. All procedures conformed to the guidelines of the International European ethical standards for the care and use of laboratory animals. All protocols were approved by the Ethical Deontological Committee for animal experimentation of the University of Pisa.

Cortical membranes for NET binding assays were prepared by homogenizing freshly dissected rabbit cerebral cortex in 30 vols of ice-cold 50 mM Tris-HCl buffer, pH 7.4, containing 120 mM NaCl and 5 mM KCl (T1 buffer). The homogenate was centrifuged at 48,000*g* for 10 min at 4 °C. The resulting pellet was suspended in T1 buffer, incubated at 37 °C for 10 min to remove endogenous norepinephrine, and centrifuged at 48,000*g* for 10 min at 4 °C. This washing procedure was repeated twice. The resulting pellet was immediately used in the binding assay or frozen at −80 °C until the time of the assay.

Membranes used in DAT and SERT binding assays were prepared from frozen rabbit *striatum* and frontal cortex as previously described.^29^

#### Separation of Human Platelets and Membrane Preparation

Venous blood (20 mL) was collected from healthy human subjects and gently mixed with 1 mL of anticoagulant (0.15 M EDTA). Platelet-rich plasma was obtained by low-speed centrifugation (200*g* for 20 min at 22 °C). Platelets were counted automatically with a flux cytometer (Cell-dyn 3500 system; Abbott, Milano, Italy). Written consent was obtained from all subjects, and the study was approved by the local Ethics Committee.

For measurement of the [^3^H]5-HT uptake, platelets were used immediately; whereas for [^3^H]paroxetine binding, platelets were precipitated by centrifugation at 10,000*g* for 10 min at 4 °C, and the pellets were then stored at −80 °C until the assay.

For human platelet membrane preparation, platelet pellets were washed with 10 mL of 50 mM Tris-HCl buffer, pH 7.4, containing 150 mM NaCl and 20 mM EDTA. Pellets were lysed and homogenized in 10 mL of 5 mM Tris-HCl buffer, pH 7.4, containing 5 mM EDTA and protease inhibitors (200 μg/mL bacitracin, 160 μg/mL benzamidine, and 20 μg/mL soybean trypsin inhibitor) using an Ultra-Turrax homogenizer and centrifuged at 48,000*g* for 15 min at 4 °C. The resulting pellets were resuspended in 50 mM Tris-HCl buffer, pH 7.4, containing 120 mM, NaCl, and 5 mM KCl (assay buffer). Protein concentration was determined according to the method of Lowry et al.^59^ after solubilization in 0.75 M NaOH and using bovine serum albumin (BSA) as standard.

#### Radioligand Binding Studies

##### [^3^H]Nisoxetine Binding Assay to Rabbit Cortical Membranes

For NET binding assays, [^3^H]nisoxetine binding was performed essentially as described by Tejani-Butt et al.^60^ The cortical membrane pellet was resuspended in 50 mM Tris-HCl buffer, pH 7.4, containing 300 mM NaCl and 5 mM KCl (T2 buffer). The binding assay was performed incubating aliquots of membranes (0.2–0.3 mg of protein) in T2 buffer with 1 nM [^3^H]nisoxetine (specific activity, 80 Ci mmol^–1^; PerkinElmer Life Science) in a final volume of 0.5 mL. Incubation was carried out at 4 °C for 4 h. Nonspecific binding was defined in the presence of 10 μM desipramine. Specific binding was obtained by subtracting nonspecific binding from total binding and approximated to 85–90% of total binding. The binding reaction was quenched by filtration through Whatman GF/C glass-fiber filters using a Brandel Harvester. Filters were washed four times with 5 mL of the ice-cold binding buffer and placed in vials with 4 mL of a scintillation cocktail. Radioactivity was measured by means of a β-counter.

NET binding parameters (maximal binding capacity, *B*_max_, fmol/mg protein; dissociation constant, *K*_d_, nM) were evaluated in rabbit cortical membranes by measuring specific binding of [3H]nisoxetine at increasing concentrations of the radioligand.

##### [^3^H]WIN 35,428 Binding Assay to Rabbit Striatal Membranes

DAT binding assays were performed using 2 nM [^3^H]WIN 35,428 (specificity activity, 84.5 Ci mmol^–1^; PerkinElmer Life Science) as previously described by Nencetti et al.^29^

##### [^3^H]Paroxetine Binding Assay to Rabbit Cortical Membranes

SERT binding assays were performed using 0.1 nM [^3^H]paroxetine (specificity activity, 15–20 Ci mmol^–1^; PerkinElmer Life Science) as previously described.^29^

##### [^3^H]Paroxetine Binding Assay to Human Platelet Membranes

For SERT binding assays, human membranes preparations and [^3^H]paroxetine (specific activity, 19 Ci mmol^–1^; PerkinElmer Life Science) were incubated as previously described by Giannaccini et al.^61^ Platelet membrane pellets were resuspended in assay buffer, and the binding assay was performed incubating aliquots of membranes (0.05–0.1 mg of protein) in a final volume of 2 mL of assay buffer. Incubation was carried out at 22 °C for 1 h. Nonspecific binding was defined in the presence of 10 μM fluoxetine. Specific binding was obtained by subtracting nonspecific binding from total binding and approximated to 85–90% of total binding. The binding reaction was quenched by filtration through Whatman GF/C glass-fiber filters using a Brandel Harvester. Filters were washed four times with 5 mL of the ice-cold binding buffer and placed in vials containing 4 mL of a scintillation cocktail. Radioactivity was measured by means of a β-counter.

SERT binding parameters (maximal binding capacity, *B*_max_, fmol/mg protein; dissociation constant, *K*_d_, nM) were evaluated in human platelet membranes by measuring specific binding of [^3^H]paroxetine at increasing concentrations of the radioligand.

Compounds (stock solutions 1 mM) were routinely dissolved in ethanol and then diluted in Tris-HCl assay buffer at the required concentration. Competition binding assays were performed using at least seven different compound concentrations, which spanned 3 orders of magnitude and approximately adjusted for the IC_50_ value of each compound. The concentration of tested compounds which produced 50% inhibition of specific [^3^H]paroxetine binding (IC_50_ values) was computer-generated using a nonlinear regression analysis of the GraphPad Prism, Version 5.0, program (GraphPad Prism, Inc., San Diego, CA). The IC_50_ values were converted to inhibition constants values (*K*_i_) using the Cheng and Prusoff^62^ equation, *K*_i_ = IC_50_/([L]/*K*_d_, where [L] is the ligand concentration. The *K*_d_ of [^3^H]paroxetine binding to human platelet membranes was 0.08 ± 0.02 nM.

##### Functional Studies: The [^3^H]5-HT Uptake to Human Platelets

The [^3^H]5-HT uptake was performed in human platelets as described by Bazzichi et al.^63^ Briefly, aliquots of platelets (2 × 10^6^ platelets) were incubated for 10 min at 37 °C with six different concentrations (15 to 700 nM) of [^3^H]5-HT (specific activity, 30 Ci mmol^–1^; PerkinElmer Life Science) in a 0.5 mL final volume of 1.17 mM KH_2_PO_4_/25 mM NaHCO_3_, pH 7.4, buffer containing 118 mM NaCl, 4.7 mM KCl, 1.07 mM MgSO_4_, 11.6 mM glucose, 0.1% ascorbate, and 100 μM pargyline. A nonspecific uptake was measured in the presence of 10 μM fluoxetine. A specific uptake was obtained by subtracting the nonspecific uptake from the total uptake and approximating it to be 85–90% of the total uptake. The uptake reaction was quenched by filtration through Whatman GF/C glass-fiber filters using a Brandel Harvester (see above). Filters were washed four times with 5 mL of the ice-cold reaction buffer and placed in vials with 4 mL of a scintillation cocktail. Radioactivity was measured by means of a β-counter (see above).

The maximal uptake rate of SERT (*V*_max_, pmol/10^9^ cells per minute) and the Michaelis–Menten constant (*K*_m_, nM) were determined in saturating conditions by increasing [^3^H]5-HT concentration. The *V*_max_ and *K*_m_ values were obtained by direct weighted nonlinear regression analysis of uptake rates against [^3^H]5-HT concentrations using the GraphPad Prism, Version 5.00, program (GraphPad Prism, Inc., San Diego, CA).

Compounds were dissolved in ethanol to obtain 1 mM stock solutions and then diluted in a Tris-HCl saline buffer at the required concentrations. In the assay, ethanol never exceeded 0.5%. Using a saturating concentration of [^3^H]5-HT (25 nM) and increasing concentrations (0.01 to 1,000 nM) of the compounds, we initially evaluated the percentage inhibition of the [^3^H]5-HT uptake. The IC_50_ value of each tested compound was computer-generated using a nonlinear regression analysis of the GraphPad Prism program (Version 5.00). To obtain compound *K*_i_ values, the apparent *K*_m_ of the [^3^H]5-HT uptake in the presence of fixed inhibitor concentrations was determined using the double-reciprocal Lineweaver–Burk plot, which also allowed for verification of the type of inhibition.

## Supporting Information Available

Figure S1, multiple sequence alignment of transporters; Figure S2, superposition of dDAT and hDAT; Figure S3, comparison between intracellular loop 5 of hDAT and dDAT; Figure S4, docking of RTI-113 and RTI-229 in hDAT, hSERT, and hNET; Figure S5, docking of maprotiline, indalpine, and zimelidine in hDAT, hSERT, and hNET; Figure S6, contour maps of PLS pseudocoefficient plots in region of Phe323 of hNET model; and Figure S7, experimental residuals versus predicted activities of compounds **1–19**, **20a–d**, and **21a–d** against SERT (PDF)
