# Supplementary material for: Focus on Human Monoamine Transporter Selectivity. New Human DAT and NET Models, Experimental Validation, and SERT Affinity Exploration
Source: ACS Chem Neurosci. 2020 Sep 29;11(20):3214–32. doi: 10.1021/acschemneuro.0c00304 (PMC8015229; doi:10.1021/acschemneuro.0c00304)
Supplement: Supplementary file 1 — cn0c00304_si_001.pdf [file cn0c00304_si_001.pdf]

Focus on Human Monoamine Transporters Selectivity. New Human DAT and NET Models, Experimental Validation and SERT Affinity Exploration.

Gabriella Ortore[1,*], Elisabetta Orlandini[2,3], Laura Betti[1], Gino Giannaccini[1], Maria Rosa Mazzoni[1], Caterina Camodeca[1] and Susanna Nencetti[1,*]

1. *Department of Pharmacy, University of Pisa, Via Bonanno 6, 56126, Pisa, Italy.*
2. *Research Center "E. Piaggio", University of Pisa, Pisa, 56122, Italy*
3. *Department of Earth Sciences, University of Pisa, Via Santa Maria 53-55, 56100, Pisa, Italy*

Supporting Information: this section contains supplementary figures.
Figure S1, multiple sequence alignment of transporters; Figure S2, superposition of dDAT and hDAT; Figure S3, comparison between intracellular loop 5 of hDAT and dDAT; Figure S4, Docking of RTI-113 and RTI-229 in hDAT, hSERT and hNET; Figure S5, docking of maprotiline, indalpine and zimelidine in hDAT, hSERT and hNET; Figure S6, contour maps of the PLS pseudocoefficient plots in the region of Phe323 of the hNET model; Figure S7, experimental residuals versus predicted activities of compounds **1-19**, **20a-d**, **21a-d** against SERT.

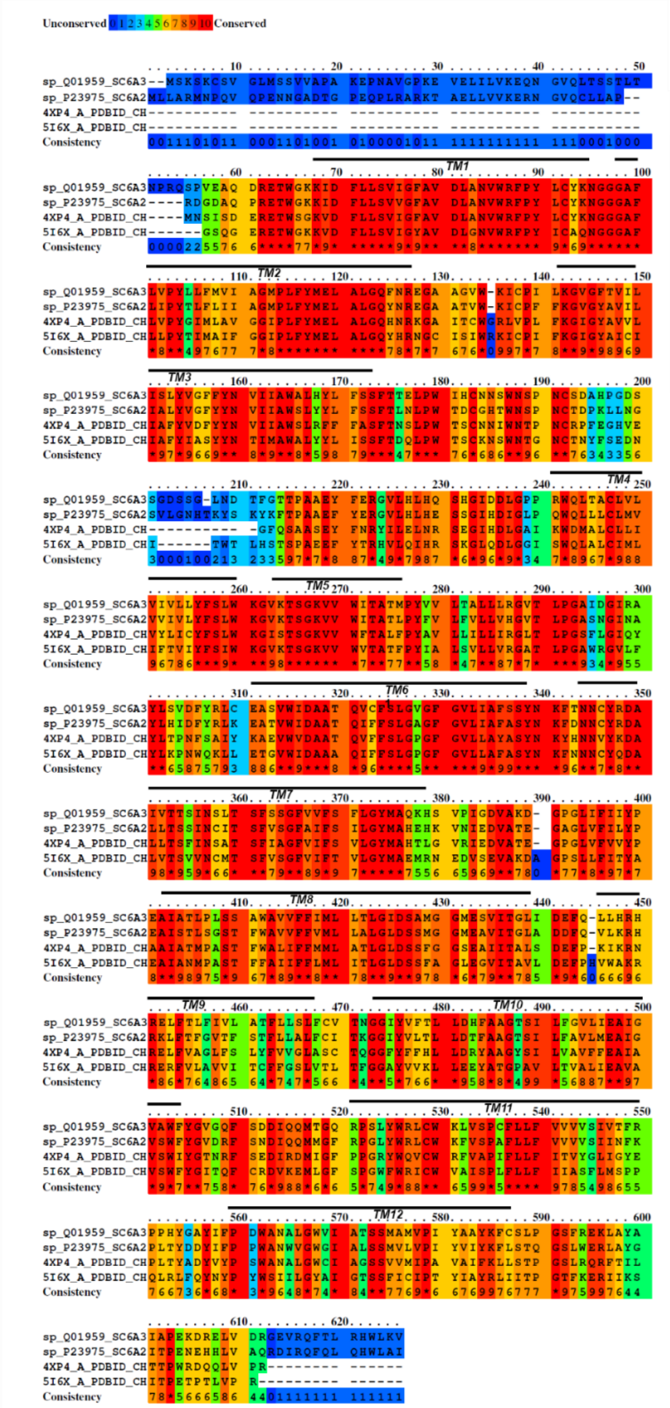

Figure S1: Multiple sequence alignment of hDAT and hNET on dDAT (4XP4 PDB code) and hSERT (5I6X PDB code) sequences used as templates. Transmembrane sequence tracts are labelled with a black line.

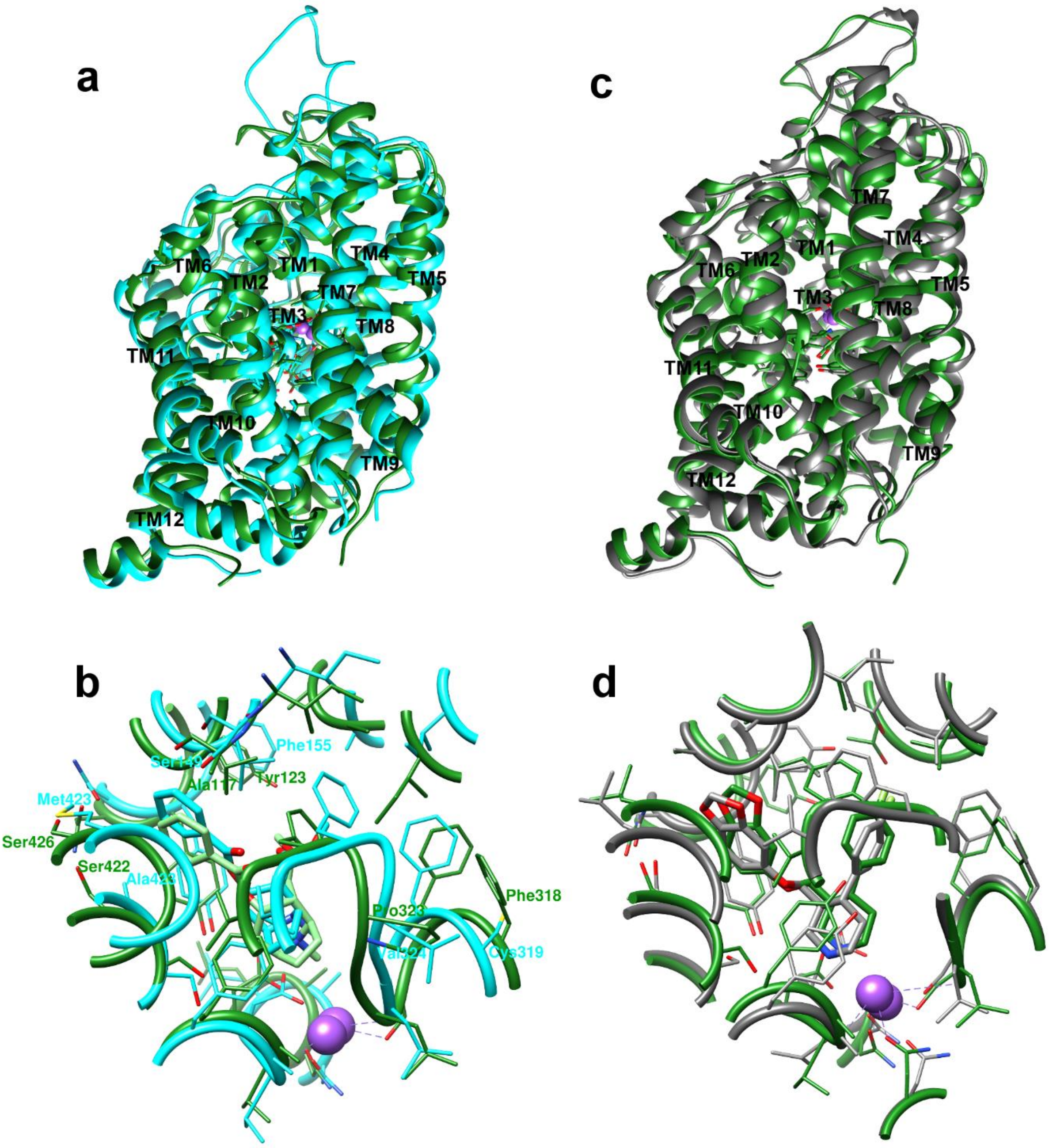

Figure S2. Superposition of the hDAT final model (cyan colored) on the dDAT template (green colored): a) overall superposition, b) binding site superposition. Superposition of the *wt*-hSERT final model (gray colored) on the hSERT template (green colored): c) overall superposition, d) binding site superposition.

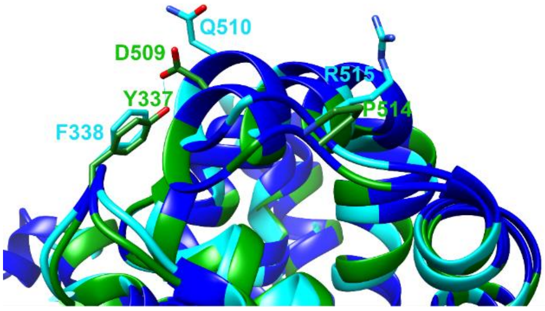

Figure S3. Comparison between intracellular loop 5 (IL5 loop) of hDAT model (cyan colored) and dDAT template (green colored)

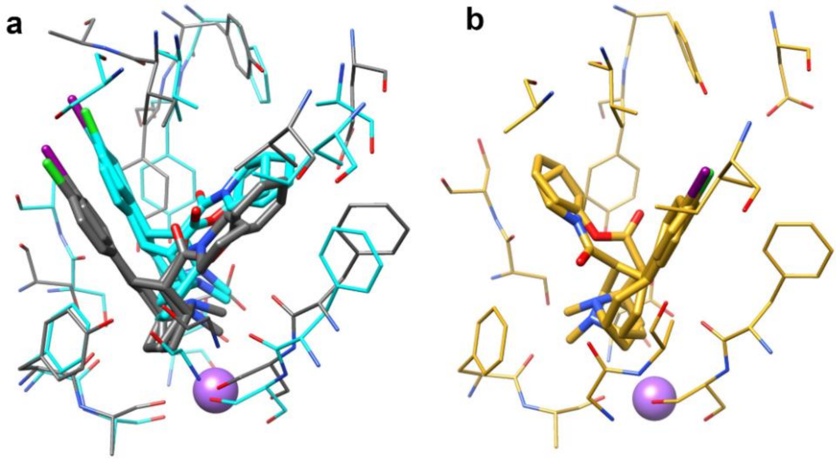

Figure S4. Docking of RTI-113 and RTI-229 in hDAT (cyan colored) and hSERT (gray colored) (a), and in hNET model (b)

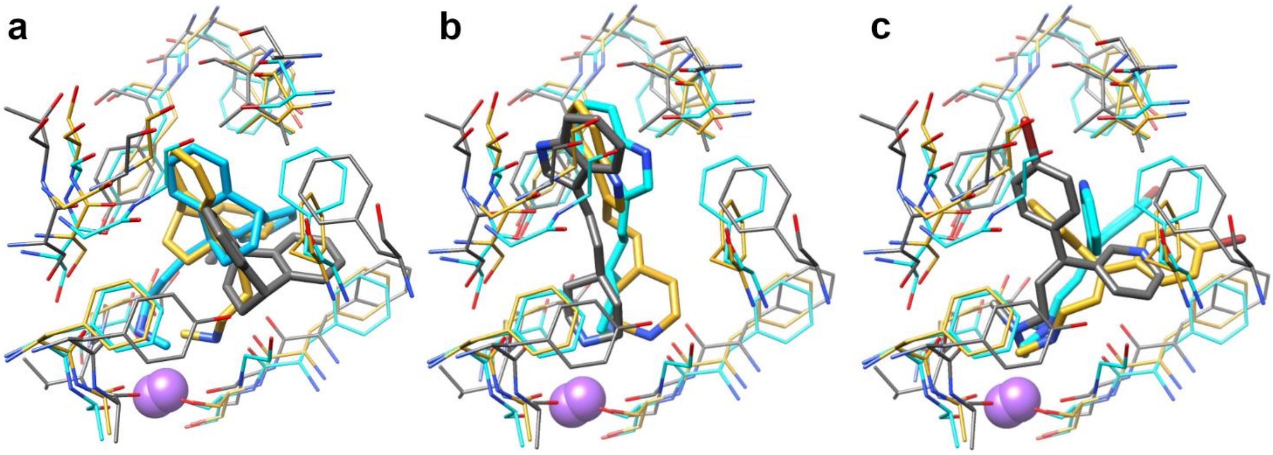

Figure S5. Docking of maprotiline (a), indalpine (b) and zimelidine (c) in hDAT (cyan colored), hNET (b) hSERT (gray colored) models.

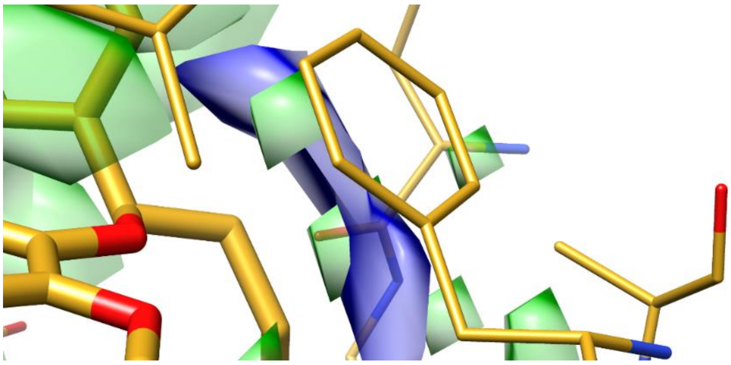

Figure S6: Contour maps of the PLS pseudocoefficient plots obtained with the NH probe at lower energy level (blue), and DRY probe at higher energy level (green), superposed on the hNET model in the region of Phe323

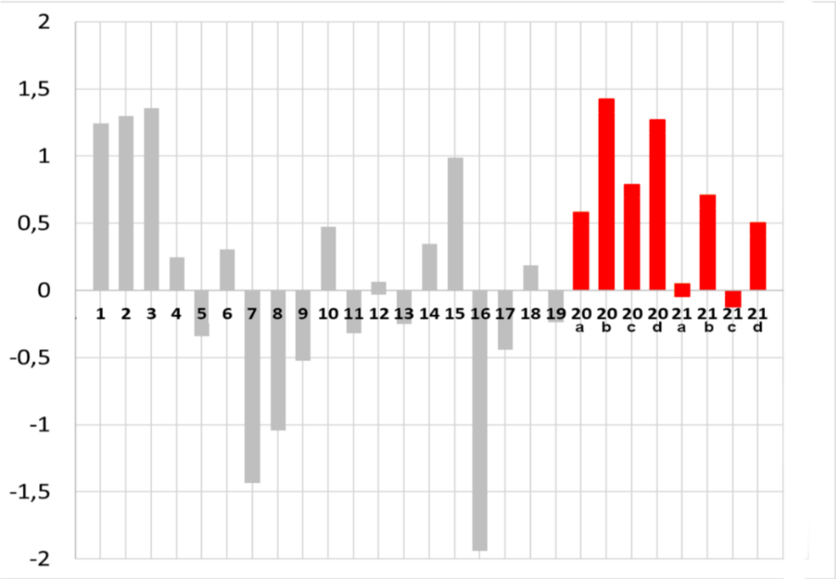

Figure S7: Experimental residuals versus predicted activities of compounds **1-19**, **20a-d**, **21a-d** against SERT. Gray colored the training set compounds tested in human and rat tissue, red colored the external test set compounds tested in rabbit membranes.
